# Mutant allele quantification reveals a genetic basis for TP53 mutation-driven castration resistance in prostate cancer cells

**DOI:** 10.1038/s41598-018-30062-z

**Published:** 2018-08-21

**Authors:** Kefeng Lei, Ran Sun, Lee H. Chen, Bill H. Diplas, Casey J. Moure, Wenzhe Wang, Landon J. Hansen, Yulei Tao, Xufeng Chen, Chin-Pu Jason Chen, Paula K. Greer, Fangping Zhao, Hai Yan, Darell D. Bigner, Jiaoti Huang, Yiping He

**Affiliations:** 1 Department of Pathology, Duke University Medical Center, Durham, NC 27710 USA; 2 The Preston Tisch Brain Tumor Center, Duke University Medical Center, Durham, NC 27710 USA; 3 General Surgery, Zhejiang Provincial People’s Hospital, Hangzhou Medical College, Zhejiang, 310014 China; 4 Scientific Research Center, China-Japan Union Hospital, Jilin University, Jilin, 130033 China; 5 Center for Molecular Medicine, Zhejiang Academy of Medical Sciences, Hangzhou, Zhejiang, 310012 China; 6 Genetron Health, Durham, NC 27709 USA

## Abstract

The concept that human cancer is in essence a genetic disease driven by gene mutations has been well established, yet its utilization in functional studies of cancer genes has not been fully explored. Here, we describe a simple genetics-based approach that can quickly and sensitively reveal the effect of the alteration of a gene of interest on the fate of its host cells within a heterogeneous population, essentially monitoring the genetic selection that is associated with and powers the tumorigenesis. Using this approach, we discovered that loss-of-function of TP53 can promote the development of resistance of castration in prostate cancer cells via both transiently potentiating androgen-independent cell growth and facilitating the occurrence of genome instability. The study thus reveals a novel genetic basis underlying the development of castration resistance in prostate cancer cells and provides a facile genetic approach for studying a cancer gene of interest in versatile experimental conditions.

## Introduction

Germline or somatic mutations occur constantly at a measurable rate in the human body^1–3^. Frequently, mutations in the human genome do not disturb the net balance of cell numbers (i.e., cell death versus cell birth). However, mutations providing proliferation/survival advantage to their host cells can achieve expansion, in which the host cells propagate, shift the balance, and eventually become clonal (e.g., driver mutations occurring in the earliest stage), or sub-clonal (e.g., driver mutations occurring in later stages) such that it is feasible for them to be identified as cancer genes^4^. Two applications that arise from this conception are: *(i)* decoding of the human cancer genome that leads to identification of most, if not all, critical genes whose mutations drive the development of human cancer, an area of research that has been extremely important and fruitful^4,5^; and *(ii)* a challenging task of functional studies of cancer genes via genetically modifying them (i.e., recapitulating their alterations in cancers) in appropriate experimental contexts^6–8^.

This latter implication, frequently via somatic gene targeting, has become an increasingly common pursuit, largely powered by new genome editing technologies such as CRISPR^6,9^. One straightforward strategy for utilizing somatic gene targeting is to generate isogenic, clonal cell lines that carry specific alterations in a gene of interest, an approach that has provided much insight into cancer gene function in the past two decades^6,10^. However, generating such isogenic cell lines may not be readily feasible for genetic alterations that result in cell growth retardation or cell lethality^11^. Even for non-damaging alterations, the process of generating isogenic cell lines can be complicated and laborious^8^. These challenges are further compounded by the fact that many cancer genes function in a cellular context-dependent manner, thus necessitating their functional assessment in multiple cell models. Another strategy, the recently developed CRISPR library-based screening and barcoding-based editing monitoring approaches, has been demonstrated to be a powerful approach for functional screenings of cancer genes in both cell lines and in animal models, although it frequently requires next generation sequencing and more sophisticated designs and analyses^12–15^. For most functional studies of a cancer gene of interest, however, a facile genetic-targeting approach with rapid readouts can be extremely helpful. Here, we describe such a genetic approach and use it to reveal the unique role of TP53’s loss-of-function in the development of castration-resistant prostate cancer (CRPC).

## Results

### Establishing and validating the Gene Editing - Mutant Allele Quantification approach

**Figure 1:**
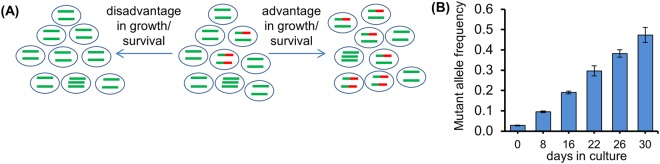
Gene Editing – Mutant Allele Quantification. **(A)** Gene mutation-driven cell evolution leads to altered allele frequencies of the mutated gene. Red color denotes mutations. **(B)** Validating gene editing- mutant allele quantification (GE-MAQ) using isogenic pairs of cell lines with or without carrying mutant *PPM1D* alleles. The parental HCT116 cells (*PPM1D*^+/*mut*^) were mixed with the isogenic HCT116 (*PPM1D*^+/+^) at 1:10 ratio, and the mixed cells were cultured under standard culture condition (and split whenever a confluence was reached). A fraction of mixed cells was taken at each indicated time point for genomic DNA (gDNA) preparation and mutant allele quantification.

We have devised an effective assay, termed Gene Editing - Mutant Allele Quantification (GE-MAQ), which can be used to readily monitor the effect of a cancer gene’s gain- or loss-of-function on cell propagation in desired experimental contexts. The basis for this approach is to simulate a pre-existing genetic alteration-driven tumorigenesis by measuring the relative abundance of alleles of interest so that the relative abundance of cells bearing those alleles under desired culturing conditions can be precisely determined and monitored (Fig. 1A). To initially establish the proof-of-principle of this approach, we took advantage of human cancer cell lines that carry a gain-of-function mutant PPM1D gene (the parental cell line; PPM1D+/mut), or the slower growing, derivative isogenic lines that carry only wild-type alleles (*PPM1D*^+/+^)^16^. We designed a locked nucleic acid (LNA) primer-based polymerase chain reaction (PCR) procedure for amplifying specifically the mutant PPM1D allele (Fig. S1a). As expected, when the parental cells were co-cultured as a minor population with the *PPM1D*^+/+^ derivative line for an extended period of time, the relative abundance of the mutant PPM1D alleles increased such that by the end of three weeks, the relative frequency of the mutant *PPM1D* allele approached that of a pure parental culture, suggesting a complete takeover of the faster-growing parental cell line in the cultures (Fig. 1B, and Fig. S1b).

**Figure 2:**
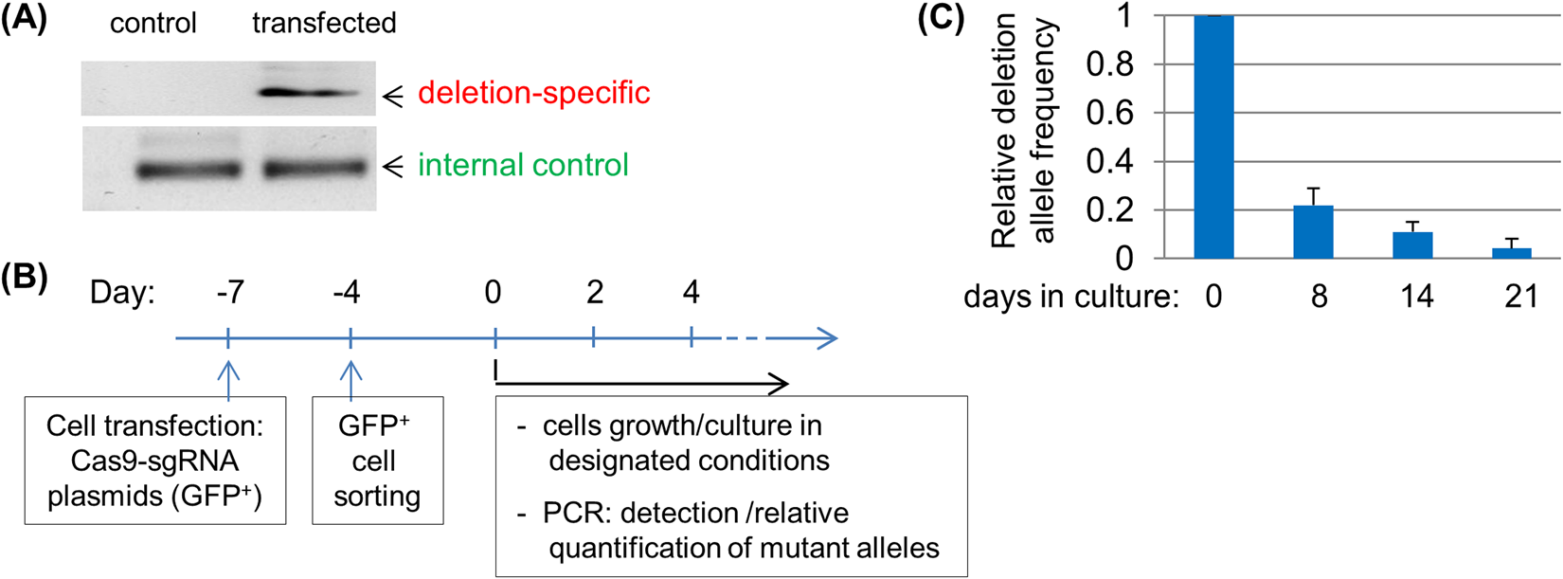
*KMT2D* inactivation abrogates propagation of prostate cancer cells. **(A)** Detection of CRISPR-induced deletion in KMT2D alleles. LNCaP cells were transfected with control Cas9 only, or Cas9 plus KMT2D-specific sgRNAs. gDNA was used for polymerase chain reaction (PCR) detection of knockout-specific and control amplicon. **(B)** Experimental design for generating and monitoring the mutant alleles. **(C)** LNCaP cells were cultured post-cell sorting, and gDNA was prepared at different time points for relative quantification of the KMT2D alleles with designated deletion.

We then tested GE-MAQ in studying the consequence of the loss-of-function of KMT2D (a.k.a. MLL2/MLL4), a gigantic epigenetic regulator gene that has been found to have mutations in a variety of human cancers. Generating clonal isogenic cell lines via somatic gene engineering is a particularly useful approach for studying *KMT2D*, as its massive size complicates an exogenous expression, gain-of-function strategy^17^. However, this approach is complicated by two problems: the highly cellular-context dependent role of KMT2D’s mutations, and the suggestion that its inactivation leads to genomic instability^11,18,19^, both of which can be overcome by generating a *KMT2D* knockout population. We designed a pair of CRISPR-based sgRNA that flank the enzymatic SET domain coding region of the *KMT2D* gene so that targeted alleles carrying deletions, via the action of both sgRNAs, can be sensitively detected (Figs S2a and S2b). When CRISPR-transfected populations of HEK293 cells, containing a mixture of various modified *KMT2D* alleles, including those with designated deletions, were mixed with non-transfected cells at various ratios, semi-quantitative PCR analysis of the relative abundance of the alleles with deletions accurately matched the fractions of the cells harboring those alleles (Fig. S2c). We applied GE-MAQ to two established human cell lines (LNCaP and LAPC-4) that originated from prostate cancer. As expected, transient delivery of CRISPR induced readily detectable KMT2D alleles with designated deletions, suggesting an unspecified sub-population of cells carrying those alleles in the population (Fig. 2A and Fig. S3a). Remarkably, the relative frequencies of such modified alleles steadily declined as the cultures were allowed to expand in extended periods of time, suggesting cells bearing deletion alleles were selected against temporally, likely due to their compromised proliferation caused by the loss of KMT2D activity as previously described^11,20^ (Fig. 2B,C and Fig. S3b). We also tested the fate of cells bearing inactivating mutations other than the designated deletions, including but not limited to those small, out-of-frame indels induced by single sgRNA, by analyzing an amplicon spanning the upstream sgRNA-targeted site (Fig. S3c). As expected, a reduction in the frequency of mutant alleles (e.g., those with small indels) was observed as revealed by surveyor analysis of the earliest and subsequent time points (Fig. S3d). Together, these results support previous findings that KMT2D is important for the propagation of established human cancer cells and further validate GE-MAQ^11,20^.

### Establishing the GE-MAQ assay for studying TP53 in prostate cancers

We applied GE-MAQ to study TP53 in the context of prostate cancer, the most common malignancy in men. Primary or metastatic prostate cancer, when presented as hormone-dependent cancer, can be effectively treated by hormone therapy; however, resistance ultimately occurs, leading to a challenging clinical problem in the form of rapidly lethal CRPC. The TP53 mutation was found to be the most significantly enriched genetic event in CRPC compared to hormone-sensitive stages of cancer^21^, which led us to hypothesize that the TP53 mutation is a powerful factor in driving the development of castration resistance in prostate cancer.

To test this hypothesis, we designed a pair of sgRNAs targeting TP53 to achieve inactivating deletions in the TP53 allele (Fig. S4a). We used the LNCaP cell line as a model because it is an established cell line that carries wild-type TP53 alleles and is sensitive to androgen depletion^22^. The deletion-specific fragment can be readily detected in host cell lines after a transient delivery of the pair of CRISPR plasmids, suggesting the generation of a heterogeneous cellular population with modified TP53 alleles, including those with inactivating deletions (Fig. S4b,c). We examined alleles bearing no designated deletions (i.e, those affected by one single sgRNA). As expected, in addition to the deletion, mutants with small indels were also generated by an individual sgRNA (Fig. S4d), suggesting the generation of a heterogeneous culture (“mutant” population).

We mixed genomic DNA (gDNA) from this mutant population with the gDNA from the parental LNCaP population with precisely determined fractions and used these mixed templates to validate the qPCR-based deletion allele frequency quantification (Fig. S5a). When this mutant population was mixed with the parental, androgen-dependent prostate cancer cell line LNCaP (at the ratio of ~1:9; named “mixed mutant” population), the relative abundance of the deletion alleles decreased proportionally, as expected (Fig. S5b). The mixture with the parental, non-transfected cells served two purposes in this case: *(i)* it provided a reliable reference for relative quantifications, and *(ii)* it ensured a reliable measurement of the relative abundance of deletion alleles to wild-type alleles by minimizing the interference from cells carrying inactivated alleles other than the designated deletion (e.g., the unspecified number of inactivating dupA allele (D48fsX51), as shown in Fig. S4d and Fig. S6a,b). The medium used in culturing these cells was the standard media with 10% regular fetal bovine serum (FBS; 100% of serum in the medium was regular FBS), providing a non- or minimal-castration condition.

### TP53 inactivation potentiates prostate cancer cells’ growth and confers an adaption to castration environments

**Figure 3:**
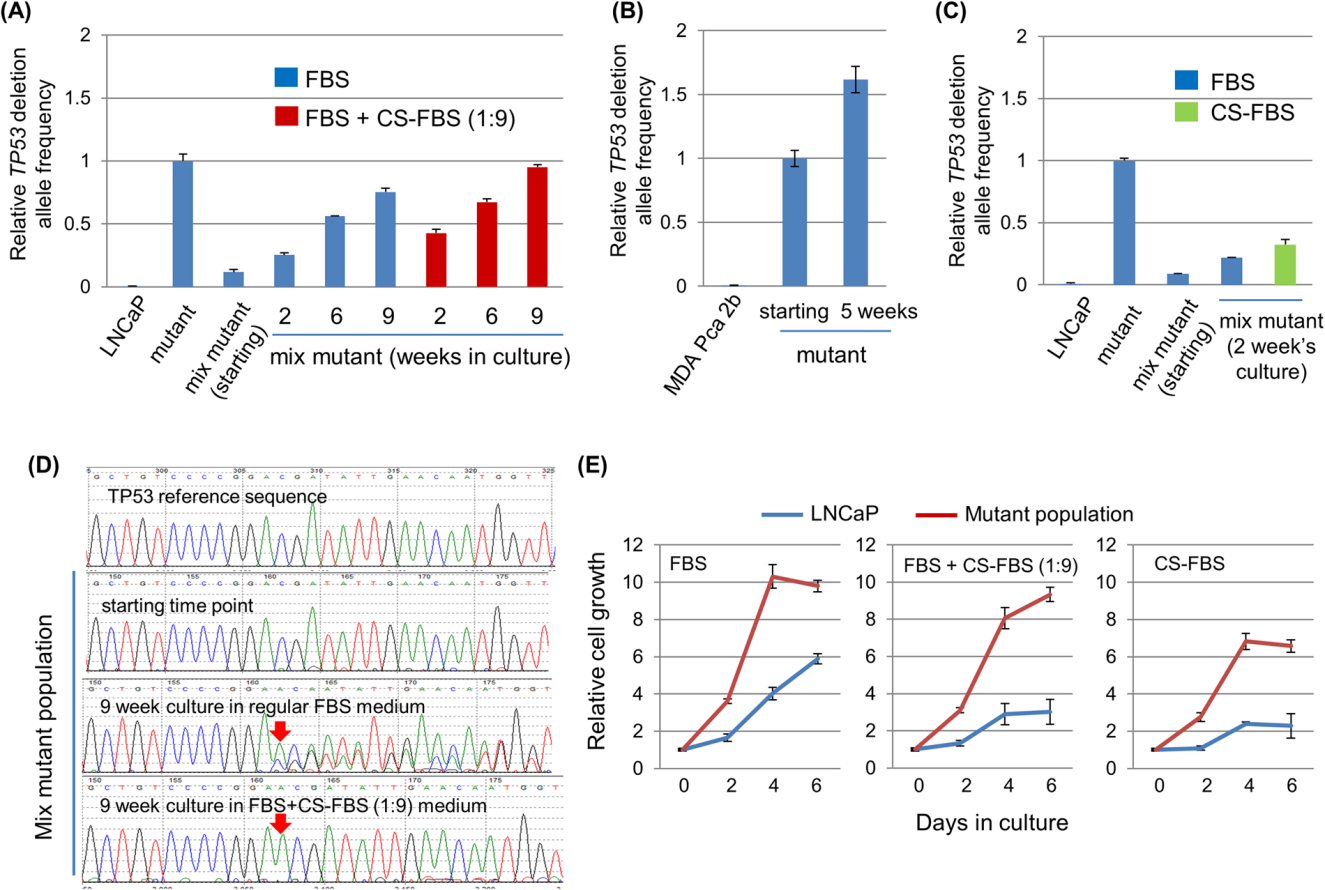
*TP53* inactivation provides an advantage to host cells under castration conditions. **(A)** *TP53* mutant population (mutant) was mixed with the parental LNCaP population (termed “mix mutant,” in which mutant made up 10% of total population). The mix mutant population was maintained either in regular fetal bovine serum (FBS)-supplied media (no castration) or in FBS/charcoal-stripped FBS (CS-FBS)-supplied media (partial castration) and split whenever a confluence was reached. A fraction of mixed cells was taken at each indicated time point for gDNA preparation and mutant allele quantification. **(B)** A similar CRISPR-mediated *TP53* mutation and GE-MAQ experiment in MDA PCa 2b cell line cultured under the standard (no castration) culture media. In this case, the starting population was the initial CRISPR-transfected, fluorescence-activated cell sorted (FACS) cells without being mixed with the parental cells. **(C)** Similar experiments with the LNCaP mix mutant population as described in (A), except the mix mutant population was maintained in regular FBS-supplied or in CS-FBS-supplied media (complete castration). **(D)** Similar experiments with the mix mutant population described in (A), except standard PCR and Sanger sequencing was performed to evaluate the small indels around sgRNA-E4 targeted site. **(E)** Proliferation of the parental LNCaP cells and the TP53 mutant population in different medium conditions as measured by a standard cell growth assay (via cell counting kit 8) in a 96-well plate.

We then investigated the fate of cells carrying TP53 allelic deletion under experimental castration conditions. Charcoal-stripped FBS (CS-FBS)-supplemented media provides a well-established condition for experimental castration^23^. As the precise experimental hormone concentrations required for experimentally mimicking patients’ situations (with or without castration) are undefined, we made up media with 10% of serum consisting of both regular FBS and CS-FBS at a ratio of 1:9 (thus, only 10% of serum in the media was regular FBS and the other 90% of serum was charcoal-stripped, providing a partial castration condition), as well as a medium with 10% of CS-FBS only (0% of regular FBS, providing a complete castration condition). We then subjected the aforementioned mixed population to the culture under the standard medium or each of the two medium conditions. To measure the full impact of different castration conditions on cell propagation, cells were split 1:6 whenever a confluence was reached. We found that even under the regular FBS media (the no-castration condition), the relative abundance of the deletion allele had increased slowly in the culture (Fig. 3A). Notably, this cell growth advantage conferred by the p53 loss-of-function under the standard, no-castration culture condition was not unique to the LNCaP cell line, as it was also observed in another *TP53*-wildtype prostate cancer cell line, MDA PCa 2b (Fig. 3B). It was also recapitulated when mixed cells were implanted *in vivo* in xenografts (Fig. S6c and Fig. S7a). More importantly, a more rapid increase was observed under the partial castration condition, in which the allele deletion frequency by the end of the nine-week culturing reached a level close to the original input mutant population, suggesting there was an even stronger growth/survival advantage provided by deletion alleles under this castration condition (Fig. 3A). In the complete castration condition, the enrichment of the deletion alleles was also detectable, even though cells barely grew under this condition during the two-week culture span, whereby the genetic selection pressure was minimal (Fig. 3C).

Three separate lines of evidence corroborate the findings from these mixed cultures/GE-MAQ assays. First, we examined the approximate frequency of TP53 alleles with inactivating small indels (i.e., targeted only by one sgRNA, thereby bearing no designated deletion) in the mutant population maintained in regular FBS medium (no castration), and found that in the longer-term culture, the inactivating small indel alleles also increased to become dominant subpopulations (Fig. S4d and Fig. S6a,b). Second, in the mutant population mix (“mutant” population mixed with the parental LNCaP cells at a 1:9 ratio), the inactivating dupA (D48fsX51) was initially not detectable, but at the end of the 9 week’s culture, it became a visible subpopulation under the regular FBS (no castration) condition and a dominant subpopulation under the FBS + Cs-FBS (partial castration) condition (1:9) (Fig. 3D, and Fig. S8). Finally, a standard cell growth assay confirmed the growth advantage of this mutant population when compared to the parental LNCaP in the regular FBS-supplemented medium; and such an advantage became even more prominent under castration media (Fig. 3E and Fig. S9). Collectively, these results suggest that *TP53* inactivation promotes tumor cells’ adaptation to and propagation in a castration microenvironment.

### P53 serves as an intrinsic barrier for prostate cancer growth

**Figure 4:**
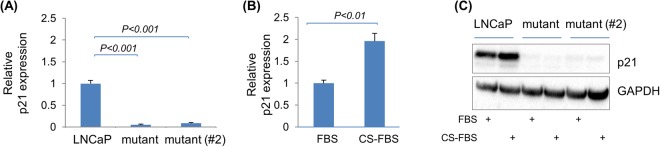
p53 activity sustains *CDKN1A* expression and responds to castration in the LNCaP cell model. **(A)** Cells were placed out in regular FBS medium. After two days, the medium was replaced with fresh, regular FBS medium; and 12 hours later, RNA was isolated for quantitative reverse transcription PCR (RT-qPCR) analysis of *CDKN1A* gene expression (*HPRT* was used as the internal control gene). **(B)** The parental LNCaP cell line was placed out in regular FBS medium. After two days, the medium was replaced with fresh, regular FBS medium or with CS-FBS-supplied castration medium; and 12 hours later, RNA was isolated for RT-qPCR analysis of *CDKN1A* gene expression (*HPRT* was used as the internal control gene). **(C)** Indicated cell lines were treated with FBS- or CS-FBS-supplied media as described in **(B)** for 24 hours and cell lysates were prepared for detection of p21 by anti-p21 immunoblot.

We investigated the mechanisms underlying the role of TP53 mutations, focusing on the two aspects described below. First, we tested the biochemical consequences of *TP53* inactivation. Most CRPC cases involve the functions of androgen receptor (AR) and/or its variants, and *AR* is the second most enriched mutated (i.e., point mutations and/or amplifications) gene in CRPC, showing more frequent aberrations compared to primary prostate cancer^21,24^. We first ruled out that the proliferation advantage observed was not due to *AR* amplification in the mutant population due to the CRISPR’s off-target effect (Fig. S10). We then tested whether *TP53* inactivation acted through a direct influence on AR signaling. We analyzed the expression of AR’s direct transcriptional targets and found no detectable increase in their mRNA levels in the mutant populations when compared to the parental LNCaP population *in vitro* and *in vivo* (indeed, in the case of *PSA* in the *in vivo* xenograft, a reduced expression level was observed) (Fig. S7b,c and Fig. S11a–c), suggesting that loss-of-function of TP53 does not directly potentiate AR’s transcriptional activity and/or its responsiveness to its ligand. To ascertain the functionality of the endogenous *TP53* in the LNCaP cell line, we measured the expression of its canonical transcriptional target, *CDKN1A*, and found that *CDKN1A* transcript is readily detectable, and most importantly, its expression is mostly abolished in the mutant populations *in vitro* and *in vivo* (Fig. 4A, Fig. S7b,c and Fig. S11d). We found that the exposure to CS-FBS medium condition promptly induced a transient upregulation of *CDKN1A* expression in LNCaP cells; while in the *TP53*-mutant populations, its expression level remained mostly attenuated (Fig. 4B,C and Fig. S11e). While the expression of *CDKN1A* can be regulated via both TP53-dependent and TP53-independent/cell cycle-dependent mechanisms^25^, and the dynamic between *CDKN1A* expression and cell cycle progression in prostate cancer is complicated^26^, these results suggest that *CDKN1A* expression in the LNCaP cell model is predominantly via the p53-dependent mechanism, and that endogenous p53 likely provides an inherent barrier to LNCaP cells’ proliferation and advancement to castration-resistant growth. Thus, TP53 loss-induced removal of such a barrier likely serves as a complementary mechanism to the recently identified double Rb1/TP53 deficiency-mediated cell lineage switch in the development of CRPC^27,28^.

### P53 inactivation facilitates genome instability in prostate cancer cells

**Figure 5:**
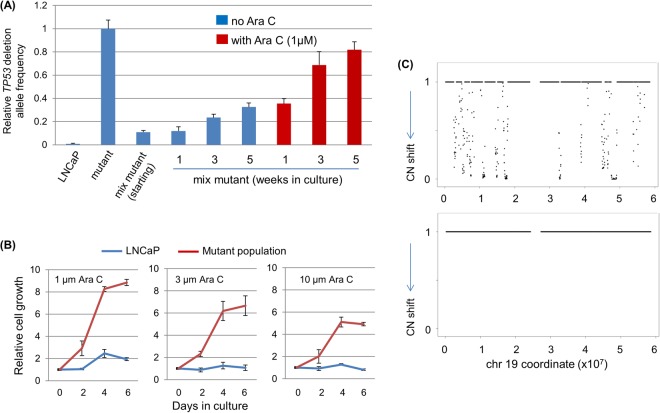
*TP53* inactivation provides a survival/proliferation advantage to host cells in the presence of Ara C. **(A)** *TP53* mutant population (mutant) was mixed with the parental LNCaP population (termed “mix mutant,” in which mutant made up 10% of total population). The mix mutant population was maintained in regular FBS-supplied media (no castration) with or without the presence of Ara C (split whenever a confluence was reached). A fraction of mixed cells was taken at each indicated time point for gDNA preparation and mutant allele quantification. **(B)** Proliferation of the parental LNCaP cells and the TP53 mutant population in regular FBS-supplied media in the presence of various concentrations of Ara C, as measured by a standard cell growth assay. **(C)** Copy number variation (CNV) analysis, as exemplified by the chr 19, in the *TP53* mix mutant population cultured in the standard medium in the presence of 1 µM Ara C for 14 weeks (top panel) and in the initial *TP53* mutant population (24-week culture, bottom panel). Each sample was compared to the parental LNCaP cell line which serves as the baseline control. The number 1 denotes no copy number change and 0 denotes copy number change regardless of gain or loss.

Next, we investigated the underlying mechanism of TP53 mutations within a genetics context (i.e., we focused on TP53 mutations’ indirect effects on the genome and genome instability). Multiple genes/pathways have been shown to contribute to the development of castration resistance, and the AR pathway is one of the most predominant among them^24^. For example, mutations and gene copy number variations (CNVs) of genes, including amplification of the AR and deletions of Rb1 and PTEN genes, are common genetic alterations in CRPC^21,29,30^. Loss of *TP53* function is one potent factor enabling CNVs upon DNA breakage^31–33^. We hypothesize that TP53 mutations can facilitate the occurrence of CNVs, thus rendering it more likely that cells with advantageous CNVs (e.g., Rb1 or PTEN deletion) will arise and be selected to become CRPC under castration conditions. In supporting this hypothesis, we found that when the aforementioned mix culture was exposed to Ara C—a cytotoxic compound that can stimulate CNV occurrence via inducing DNA damage^33,34^ —a potent protection for TP53-deficient cells was observed, as evidenced by the rapid enrichment of *TP53*’s deletion allele frequency (Fig. 5A and Fig. S12a). In consistence with this finding from the mixed population, the mutant population (without being mixed with the parental LNCaP cells) also demonstrated a resistance to Ara C treatment in comparison to the parental LNCaP, as measured by cell numbers (Fig. 5B and Fig. S12b). The propagation/survival advantage TP53-deficient cells possessed in response to a CNV-inducing agent is expected to facilitate the arising of cancer cells with additional genomic alterations such as CNVs. In supporting this scenario, a mixed culture that has been growing in regular medium with the presence of 1 µM Ara C for 14 weeks displayed heterogeneous (non-clonal) yet measurable CNVs across the genome when compared to the parental LNCaP (Fig. 5C and Fig. S12a). This heterogeneous CNV is not due to the initial side effect from the CRISPR, as it is mostly absent from the initial TP53 mutant population (Fig. 5C and Fig. S13b).

Although the experimental treatment of Ara C does not represent a real pathogenesis process and a direct test in patients is not feasible, results from aforementioned experimental models provide evidence to support the role of TP53 loss in promoting prostate cancer cells’ genome instability and the arising of androgen-independent cell growth, either via increasing the number of cells with CNVs and/or promoting a higher incidence of CNVs in a given number of cells. In either case, these findings illustrate a genetic basis underlying TP53 mutation-driven development of CRPC.

## Discussion

Our study leads us to conclude that pre-existing *TP53* mutations (i.e., loss of function) in hormone- dependent prostate cancer cells can contribute to the development of CRPC, likely via both facilitating the proliferation of and promoting the genomic instability in tumor cells. Numerous genes and genetic alterations contribute to the genesis of prostate cancers^21,29,35,36^. Results presented here provide direct evidence and a genetic basis to support a role of p53 inactivation in the transition from hormone-dependent prostate cancers to CRPC. Previous studies suggest that TP53 functionally interacts with AR, although it is unclear whether and how these interactions contribute to cancer’s progression to the castration-resistant stage^37,38^. The p53 signaling has been proposed to act in preventing the expansion of a small number of neuroendocrine (NE) lineage cells that reside in the mass of hormone-sensitive prostate carcinoma; and upon *TP53* mutation, this small subpopulation of NE cells propagates to form a rare, clinically different subtype of CRPC (small cell neuroendocrine carcinoma)^39^. Separately, the loss of TP53 together with Rb1 loss and/or PTEN mutations was recently found to shape cell lineage plasticity and reprogramming, and, by doing so, promotes cancer cells’ resistance to antiandrogen treatments^27,28^.

AR signal plays critical roles in the development and progression of CRPC and serves as a major therapeutic target. In our study, the absence of measurable TP53 loss-potentiated AR signal suggests that at least in these experimental models during the limited period of assays, AR-mediated proliferation was not a major factor in facilitating the propagation of TP53-deficient tumor cells. Instead, it is possible that the *TP53* knockout-induced down-regulation of CDKN1A contributes to and facilitates cell proliferation, given the well-established role of CDKN1A in regulating cell cycle^7,25^. While the consequences of p53 deficiency are undoubtedly multiple and profound as illustrated by these findings, our study provides a simple genetic basis that helps explain the significant enrichment of *TP53* mutations in prostate cancer’s progression to the CRPC stage. The *TP53* deficiency can facilitate the occurrence of genetic alterations and thus enlarges the pool of tumor cells that have the potential to be selected out to become CRPC, possibly through mechanisms such as direct promotion of genome instability and/or protecting cells from genomic stress/exogenous cytotoxicity agents. This model is supported by separate lines of evidence: (i) *TP53* mutation is the most significantly enriched genetic event in CRPC compared to androgen-dependent prostate cancer^21^; (ii) there is a heavier mutation burden (e.g., higher numbers of gene mutations and CNVs) in CRPC than in the earlier stage of prostate cancers^21,35^, and (iii), the role of p53 as the guardian of genome stability has been well established^40,41^. The model also speculates a scenario in which castration-resistant tumor cells exist prior to or during the castration treatment; castration-resistant tumor cells are not “induced” by castration, but the hormone therapy simply “selects” this population to become CRPC. This genetics-driven process echoes the *KRAS* mutation in providing cancer cells a propagation advantage in a hypoglycemic microenvironment^10^. The clinical implication is obvious: when treating those hormone-sensitive tumor cells with hormone therapy, simultaneous eradiation of this likely minority population of cells prior to recurrence of CRPC can be important and clinically beneficial.

Finally, we point out that this study also provides a facile genetics-based approach for studying cancer genes in versatile settings, with an obvious simplicity when compared to the recently described CRISPR barcoding-based editing monitoring approach^12,13^. The genetics-based strategy’s advantages are obvious: (i) it overcomes a potential issue with clonal variations frequently presented by the strategy of analyzing single isogenic clones, and (ii) quantifying relative allele abundance can be sensitive, efficient, and performed in a high throughput manner. While the genetic heterogeneity of cancer cells frequently presents a challenge in our study of cancer, the approach described in this study points to a way to take advantage of such heterogeneity and turn it into an asset in studying cancer gene functions.

## Methods and Materials

### Cell lines and cell culture

The human HEK293 cell line (Invitrogen, Cat# R70007) was cultured in DMEM medium supplied with 10% regular fetal bovine serum (FBS) (GIBCO, cat# 10438026). HCT116 cell lines and their maintenance were described previously^16^. The human prostatic carcinoma cell line, LNCaP (ATCC, cat# CRL-1740), was cultured in RPMI-1640 medium containing 10% of FBS, 10% of charcoal-stripped FBS (GIBCO, cat# 12676011), or 10% of the mixture of two types of FBS as necessary. The human prostatic cancer cell line MDA PCa 2b (ATCC, cat# CRL-2422) cells were cultured in BRFF-HPC1 medium (Athena Enzyme Systems, Baltimore, MD, cat# NC9970798) supplemented with 20% FBS. The LAPC4 cell line was a gift from Dr. Alan Meeker (Johns Hopkins University) and was maintained in IMDM, supplemented with 10% of FBS. All cell lines were maintained in a humidified atmosphere at 37 °C and with 5% CO_2_. Authentication of prostatic cancer cell lines was performed by the Cancer Center Isolation Facility of the Duke Cancer Institute. For monitoring HCT116 cell line’s growth, the parental (*PPM1D*^+*/mut*^) and each isogenic *PPM1D*^+/+^ cell line were mixed at a 1:10 ratio; 3 × 10^5^ of mixed cells (starting time point, defined as day 0) were cultured in a 6 cm tissue culture plate (Coring, cat# 430166) and allowed to grow. Once cells grew to confluence, three fourths of cells were harvested for genomic DNA (gDNA) preparation, and the remaining one fourth of cells were re-plated out and allowed to grow to continue the gDNA preparation/cell re-plating steps (typically every four days at the beginning of experiments, and the time interval gradually became shorter as the cells’ growth pace slowly increased due to the slow enrichment of the parental cells). For all other cell cultures involving mutant and mixed mutant populations, cells were similarly allowed to grow to confluence and passaged as necessary. At each passage, parts of cells were harvested for gDNA preparation and remaining cells were plated out for continuous culture as necessary. For cell cultures in various medium conditions, cells were plated out in regular FBS-supplied media and allowed to fully adhere for two days before media were changed to desired experimental media (e.g., those with charcoal-stripped FBS or with DNA-damaging agent Ara C).

Authentication of prostatic cancer cell lines was performed by the Cancer Center Isolation Facility of the Duke Cancer Institute. No American Type Culture Collection (ATCC) reference of the short tandem repeat (STR) profile was available for the LAPC4 line. Thus, this line’s STR profile was compared to the available marker profile that was published^22^ and was found to have 8 out of 10 matched alleles. Both LNCaP and MDA PCa 2b cell lines were found to have a complete match with ATCC’s reference of STR profiles. Derivative cultures (with *TP53* mutations) from these two cell lines were subjected to the same STR profiling analysis and were found to have complete matches with their respective parental line.

Plasmid construction and generation of mutant cell populations The CRISPR system was used for all gene edits. pSpCas9(BB)-2A-GFP (PX458) was a gift from Feng Zhang (Addgene plasmid ^#^48138). Double-stranded oligonucleotides were inserted into restricted enzyme BbsI-linearized PX458 to construct plasmids for CRISPR targeting of human *KMT2D/MLL2* and *TP53*. For transient plasmid’s transfection, plasmids (two plasmids at a 1:1 ratio for achieving the desired gene deletion/mutations) and Transfex (ATCC, cat# ACS-4005) were mixed and used for cell transfection according to manufacturer’s instructions. Three to four days after the transfection, GFP + cells were sorted via fluorescence-activated cell sorting (BD FACSVantage SE cell sorter, Duke Cancer Institute) to obtain a GFP + population. In this case, sorting served two purposes: first, it enriched transfected cells (with potential gene edits) and, more importantly, it controlled any effects Cas9’s expression had on cells. After sorting, cells were cultured for 7–10 days to allow for cell propagation and gene editing to occur, at which points (typically 10–14 days post transfection), cells were ready for experiments involving short- or long-term culture/monitoring (either as a “mutant” population without mixing with the matched parental cell line or as a “mix mutant” population that included the matched parental cell line).

Genomic DNA preparation, PCR and q-PCR experiments gDNA was extracted using QIAamp DNA Mini Kit (QIAGEN, cat# 51306) following the manufacturer’s protocol. DNA concentration and purity was assessed by Nanodrop Lite and Qubit 2.0 Fluorometer (Life Technologies, CA). Regular PCR was performed on C1000 Touch Thermal Cycler (BIO-RAD). Each PCR reaction (total of 15 µl) included 15 ng of gDNA and 0.33 µM of each primer in 1X KAPA 2 G Fast ReadyMix PCR master mix (KAPABIOSYSTEMS, cat# KK5102), using the following cycling condition: an initial denaturation of 3 min at 95 °C, followed by 33 cycles of 95 °C for denaturation (10 sec) and 60 °C for annealing/elongation (30 sec), and ended with a final extension at 72 °C (5 min). Sanger Sequencing (performed by Eton at Research Triangle Park, NC) was used for confirming expected amplicons and genotyping genes of interest.

All quantitative PCR (qPCR) was SYBR label-based and was performed on CFX96 Real-Time System (BIO-RAD). For quantifying mutant *PPM1D* using mutant allele-specific amplification, PCR reaction first underwent a pre-amplification step using the Q5 Hot Start High-Fidelity 2X Master Mix (NEB, cat# M0494L), containing 50 ng of gDNA, and 500 nM of each pre-amplification primer. The pre-amplification program used was a touchdown program with 20 cycles of amplification. Pre-amplified samples were then diluted 1:1000 in water. The diluted pre-amplified product was used as a template for allele-specific qPCR, and the KAPA 2X SYBR FAST mix (KAPABIOSYSTEMS cat KK4602) was used for the internal control qPCR. Each qPCR reaction included 5 µl of the pre-amplified template (1:1000) and 500 nM of primers. The qPCR program was 40 cycles of amplification at an annealing temperature of 68 degrees. Expression values were based on the Ct values for allele-specific qPCR and normalized to the reference of the internal control qPCR. Three technical replicates were ran for each pre-amplified sample and pure parental HCT116 gDNA and pure repaired (WT) gDNA were used as controls for each run. For relative quantification of deletion-derived fusion DNA (deletion alleles), each qPCR reaction (15 µl) included 30 or 45 ng of gDNA and 0.33 µM of each primer in 1 X KAPA SYBR FAST qPCR mix (KAPABIOSYSTEMS, cat# KK4602) using the following program: an initial denaturation of 3 min at 95 °C, followed by 35 cycles of 95 °C (10 sec) for denaturation and 62 °C (30 sec) for annealing/elongation. The qPCR data were analyzed using Bio-Rad CFX Manager 3.1. For each reaction, the melting curve was checked to confirm the specificity of the qPCR.

### RNA preparation and RT- qPCR

Total RNA was extracted using E.Z.N.A. Total RNA Kit I (OMEGA, cat# R6834–02) or quick-RNA mini prep kit (Zymo Research, cat# 11–328) following the manufacturer’s protocols. RNA concentration was determined by Nanodrop Lite Spectrophotometer (Thermo Scientific). For gene expression analysis, reverse transcription was performed to convert total RNA into single-strand cDNA using the RNA to cDNA EcoDry Premix (Clontech, cat# 639547). Subsequently, qPCR was performed following aforementioned qPCR procedure. Each reaction included cDNA template equivalent of 10 ng of total RNA, using the following program: 95 °C 3 min; 40 cycles of 95 °C 10 s, 60 °C 20 s, and 72 °C 10 s; and a final step of 72 °C 30 s. The *HPRT1* gene was used as the internal control for RT-qPCR, as this gene was shown to be a reliable internal control gene for prostate cancers^42^. Statistical significance between groups was tested by Student’s two-tailed and unpaired t-test using Graphad software (San Diego, CA).

### Oligos and primers

All oligos and primers used for the study are listed in Supplementary Table 1.

### Immunoblotting

Total proteins were extracted from cells using RIPA Buffer (Thermo Scientific, cat# 89901). Protein concentrations were determined by Pre-Diluted Protein Assay standards (Thermo Scientific, cat# NA165380) using the Pierce BCA Protein Assay Kit (Thermo Scientific, cat# RD231228). 20 μg of total protein along with NuPAGE Sample Reducing Agent (10×) (Life Technologies, cat# NP0004) and NuPAGE LDS Sample Buffer (4×) (Thermo Scientific, cat# NP0007) were loaded onto Novex NuPAGE 4–12% Bis-Tris Protein Gels (Thermo Scientific, Cat# NP0322BOX) and transferred to nitrocellulose membranes (BIO-RAD, cat# 1620090). Membranes were blocked with 5% milk in washing buffer (50 mM Tris-HCI pH 7.5, 150 mM NaCI, and 0.05% Tween 20) at room temperature for two hours. Membranes were then incubated at 4 °C overnight with anti-P53 (sc-126, Santa Cruz Biotechnology, 1:500 dilution), anti-p21 (Cell Signaling Technology, cat# 2947, 1:1000 dilution) or anti-GAPDH for two hours (Santa Cruz Biotechnology, cat# sc25778, 1:1000 dilution) at room temperature. The following secondary antibodies were used: Anti-mouse IgG, HRP-linked Antibody (Cell Signaling Technology, #7076P2, 1:5000 dilution) and Anti-rabbit IgG, HRP-linked Antibody (Cell Signaling Technology, #7074 s, 1:5000 dilution). Immunoblot bands were detected using SuperSignal West-Femto Maximum Sensitivity Substrate and Chemiluminescent Substrate (Thermo Scientific cat# RH236503A and RA227125) and scanned/quantified via ChemiDoc MP Imaging System (Bio-Rad). Full-length gel images are displayed in Fig. S14.

### Cell proliferation assay

Cell proliferation was analyzed using CCK-8 (DojinDo, cat# ck04). Cells were plated out in 96-well plates (1,500/well in 100 µl medium) and were allowed to adhere for two days before media were replaced with desired media (e.g., castration media or with DNA damaging agent Ara C). At each experimental time point, 10 μl of CCK-8 solution was added to each well and incubated for 4 hours. Plates were read at 450 nm by a multimode microplate reader (Infinite M200 PRO; Beckman).

Xenograft models All animal work was conducted in accordance with the NIH Guidelines of Care and Use of Laboratory Animals and approved by Duke Institutional Animal Care and Use Committee (IACUC/A092–16–04). Immunocompromised NSG (NOD.Cg-PrkdcscidIl2rgtm1Wjl/SzJ) mice were from The Jackson Laboratories. 2 × 10^6^ LNCaP cells (parental LNCaP, or TP53-null, mutant#1, or the mixture of the two lines with ~20% of mutant in the mixture) were suspended in 0.1 ml 1x HBSS with 50% Matrigel (Corning), and inoculated subcutaneously into the right thigh of 6–8 weeks old male mouse (two mice for each cell line or cell line mixture). The mice were sacrificed 21 days later after implantation, and tumor tissues were collected and frozen at −80 °C for gDNA or total RNA preparation. Following our implantation procedure, at the 21 day time point post implantation, the size of xenograft tumors derived from the LNCaP cell line typically ranges from 30–40 mm^3^, as determined by caliper measurements of tumor length (L) and width (W) according to the formula (L × W^2^)/2 (the sizes of xenograft tumors for the specific experiments were indicated in the legend of Fig. S7). Measurement of mutant allele frequency and relative gene expression levels were performed following the same protocol as those used for *in vitro* cell models. Separately, parts of tumor tissues were fixed with paraformaldehyde for paraffin embedding and H&E staining to pathologically confirm the generation of tumours.

Copy Number Variation analysis CNV analysis was performed using Infinium HumanCore-24 v1.0 DNA Analysis Kit (cat# WG330–2001, Illumina, San Diego, CA). For each sample, 200 ng of high quality DNA was used for experiments following the manufacturer’s Infinium HTS protocol. Briefly, the samples were denatured and amplified overnight for 20–24 hours. Fragmentation, precipitation and resuspension of the samples followed overnight incubation. After resuspension, samples were then hybridized to the Illumina Infinium Core-24 BeadChip for 16–24 hours. Finally, the BeadChips were washed to remove any unhybridized DNA and then labeled with nucleotides to extend the primers to the DNA sample. Following the Infinium HTS protocol, the BeadChips were imaged using the Illumina iScan system. The quality of data produced was checked by uploading raw data into Illumina’s Genome Studio to ensure all call rates for values of 0.98 or greater and the appropriate control graphs in Genome Studio’s Control Dashboard. Genome Studio 2.0 was used for CNV analysis. Genotyping Module 2.0 was applied and paired sample CNV analyses were calculated with the parental LNCaP cell line as the reference. Statistical confidence level of copy number in each probe was evaluated as copy number (CN) shift, where 1 denoted no copy number change and 0 denoted copy number change (gain or loss).

## Electronic supplementary material

Supplementary Information

Supplementary Table 1

## References

[1] Bielas JH, Loeb KR, Rubin BP, True LD, Loeb LA (2006). Human cancers express a mutator phenotype. Proceedings of the National Academy of Sciences of the United States of America.

[2] Bielas JH, Loeb LA (2005). Quantification of random genomic mutations. Nature methods.

[3] Araten DJ (2005). A quantitative measurement of the human somatic mutation rate. Cancer research.

[4] Vogelstein B (2013). Cancer genome landscapes. Science.

[5] Gao J (2013). Integrative analysis of complex cancer genomics and clinical profiles using the cBioPortal. Science signaling.

[6] Waldman T (2016). The Inaugural Use of Gene Editing for the Study of Tumor Suppressor Pathways in Human Cells-p21WAF1/CIP1. Cancer research.

[7] Waldman, T., Kinzler, K. W. & Vogelstein, B. p21 is necessary for the p53-mediated G1 arrest in human cancer cells. *Cancer research* **55**, 5187–5190 (1995). 7585571

[8] Rago C, Vogelstein B, Bunz F (2007). Genetic knockouts and knockins in human somatic cells. Nature protocols.

[9] Li X, Wu R, Ventura A (2016). The present and future of genome editing in cancer research. Human genetics.

[10] Yun J (2009). Glucose deprivation contributes to the development of KRAS pathway mutations in tumor cells. Science.

[11] Guo C (2013). KMT2D maintains neoplastic cell proliferation and global histone H3 lysine 4 monomethylation. Oncotarget.

[12] Guernet A, Aaronson SA, Anouar Y, Grumolato L (2016). Modeling intratumor heterogeneity through CRISPR-barcodes. Molecular & cellular oncology.

[13] Guernet A (2016). CRISPR-Barcoding for Intratumor Genetic Heterogeneity Modeling and Functional Analysis of Oncogenic Driver Mutations. Molecular cell.

[14] Wang T, Wei JJ, Sabatini DM, Lander ES (2014). Genetic screens in human cells using the CRISPR-Cas9 system. Science.

[15] Sanchez-Rivera FJ, Jacks T (2015). Applications of the CRISPR-Cas9 system in cancer biology. Nature reviews. Cancer.

[16] Zhang L (2014). Exome sequencing identifies somatic gain-of-function PPM1D mutations in brainstem gliomas. Nature genetics.

[17] Guo C (2012). Global identification of MLL2-targeted loci reveals MLL2’s role in diverse signaling pathways. Proceedings of the National Academy of Sciences of the United States of America.

[18] Kantidakis T (2016). Mutation of cancer driver MLL2 results in transcription stress and genome instability. Genes & development.

[19] Zhu J (2015). Gain-of-function p53 mutants co-opt chromatin pathways to drive cancer growth. Nature.

[20] Kim JH (2014). UTX and MLL4 coordinately regulate transcriptional programs for cell proliferation and invasiveness in breast cancer cells. Cancer research.

[21] Robinson D (2015). Integrative clinical genomics of advanced prostate cancer. Cell.

[22] van Bokhoven A (2003). Molecular characterization of human prostate carcinoma cell lines. The Prostate.

[23] Tai S (2011). PC3 is a cell line characteristic of prostatic small cell carcinoma. The Prostate.

[24] Chandrasekar T, Yang JC, Gao AC, Evans CP (2015). Mechanisms of resistance in castration-resistant prostate cancer (CRPC). Translational andrology and urology.

[25] Macleod KF (1995). p53-dependent and independent expression of p21 during cell growth, differentiation, and DNA damage. Genes & development.

[26] Lu S, Tsai SY, Tsai MJ (1999). Molecular mechanisms of androgen-independent growth of human prostate cancer LNCaP-AI cells. Endocrinology.

[27] Mu P (2017). SOX2 promotes lineage plasticity and antiandrogen resistance in TP53- and RB1-deficient prostate cancer. Science.

[28] Ku SY (2017). Rb1 and Trp53 cooperate to suppress prostate cancer lineage plasticity, metastasis, and antiandrogen resistance. Science.

[29] Grasso CS (2012). The mutational landscape of lethal castration-resistant prostate cancer. Nature.

[30] Koivisto PA, Rantala I (1999). Amplification of the androgen receptor gene is associated with P53 mutation in hormone-refractory recurrent prostate cancer. The Journal of pathology.

[31] Livingstone LR (1992). Altered cell cycle arrest and gene amplification potential accompany loss of wild-type p53. Cell.

[32] Yin Y, Tainsky MA, Bischoff FZ, Strong LC, Wahl GM (1992). Wild-type p53 restores cell cycle control and inhibits gene amplification in cells with mutant p53 alleles. Cell.

[33] Paulson TG, Almasan A, Brody LL, Wahl GM (1998). Gene amplification in a p53-deficient cell line requires cell cycle progression under conditions that generate DNA breakage. Molecular and cellular biology.

[34] Chen Y, Goz B, Kirkman L (1993). An analysis of vincristine-resistance in BHK cells pretreated with 1-beta-D-arabinofuranosylcytosine. Anticancer research.

[35] Cancer Genome Atlas Research, N. The Molecular Taxonomy of Primary Prostate Cancer. Cell 163, 1011–1025, 10.1016/j.cell.2015.10.025 (2015). 10.1016/j.cell.2015.10.025 PMC4695400 26544944

[36] Beltran, H. *et al*. Divergent clonal evolution of castration-resistant neuroendocrine prostate cancer. *Nature medicine*, 10.1038/nm.4045 (2016). 10.1038/nm.4045 PMC4777652 26855148

[37] Alimirah F (2007). Expression of androgen receptor is negatively regulated by p53. Neoplasia.

[38] Cronauer MV, Schulz WA, Burchardt T, Ackermann R, Burchardt M (2004). Inhibition of p53 function diminishes androgen receptor-mediated signaling in prostate cancer cell lines. Oncogene.

[39] Chen H (2012). Pathogenesis of prostatic small cell carcinoma involves the inactivation of the P53 pathway. Endocrine-related cancer.

[40] Lane DP (1992). Cancer. p53, guardian of the genome. Nature.

[41] Vogelstein B, Lane D, Levine AJ (2000). Surfing the p53 network. Nature.

[42] Ohl F (2005). Gene expression studies in prostate cancer tissue: which reference gene should be selected for normalization?. Journal of molecular medicine.

