# Supplementary material for: Mutant allele quantification reveals a genetic basis for TP53 mutation-driven castration resistance in prostate cancer cells
Source: Sci Rep. 2018 Aug 21;8:12507. doi: 10.1038/s41598-018-30062-z (PMC6104024; doi:10.1038/s41598-018-30062-z)
Supplement: Supplementary file 1 — Supplementary Information [file 41598_2018_30062_MOESM1_ESM.pdf]

Mutant allele quantification reveals a genetic basis for TP53 mutation-driven castration resistance in prostate cancer cells

Kefeng Lei[1,2,3,#], Ran Sun[1,2,4, #], Lee H. Chen[1,2,#], Bill H. Diplas[1,2], Casey J. Moure[1,2], Wenzhe Wang[1,2,5], Landon J. Hansen[1,2], Yulei Tao[1], Xufeng Chen[1], Chin-Pu Jason Chen[1,2], Paula K. Greer[1,2], Fangping Zhao[6], Hai Yan[1,2], Darell D. Bigner[1,2], Jiaoti Huang[1], Yiping He[1,2,*]

[1]Department of Pathology, Duke University Medical Center, Durham, NC, 27710
[2]The Preston Tisch Brain Tumor Center, Duke University Medical Center, Durham, NC, 27710
[3]General Surgery, Zhejiang Provincial People's Hospital, Hangzhou Medical College, Zhejiang, China 310014
[4]Scientific Research Center, China-Japan Union Hospital, Jilin University, Jilin, China 130033
[5]Center for Molecular Medicine, Zhejiang Academy of Medical Sciences, Hangzhou, Zhejiang, China 310012
[6]Genetron Health, Durham, NC, 27709

[#]These authors contributed equally to this work.

[*] Corresponding author: Yiping He
Department of Pathology and The Preston Tisch Brain Tumor Center
Duke University Medical Center, Durham, NC, 27710
Email: yiping.he@duke.edu; Phone: 919-684-4760

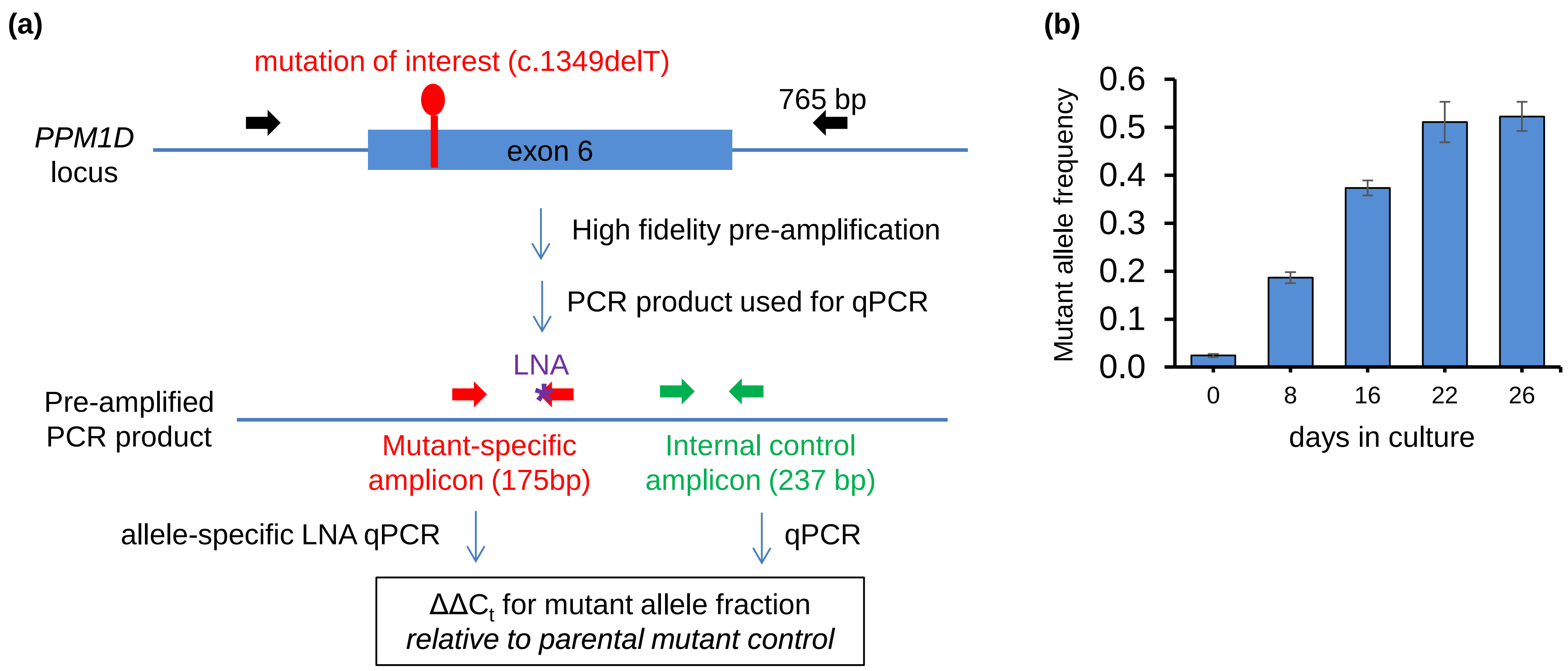

Fig. S1. Quantification of mutant *PPM1D* reveals the proliferation advantage of HCT116 cells bearing the mutant alleles. (a) A locked nucleic acid (LNA) primer-based polymerase chain reaction (PCR) procedure for amplifying specifically the mutant PPM1D allele. HCT116 harbors a 1 bp deletion (c1349delT, L450X) in *PPM1D.* We used allele-specific PCR to preferentially amplify this mutation by modifying the 3' end of the primer with LNAs, greatly enhancing its specificity. To reliably detect and quantify the mutation, we first pre-amplify the genomic DNA of the pooled cell population with a high-fidelity polymerase. After diluting the PCR product, we then perform allele-specific qPCR and a separate internal control qPCR, enabling quantification of the mutant allele fraction relative to the parental control ($\Delta\Delta C_t$). (**b**) Validating GE-MAQ using isogenic pairs of cell lines with or without carrying mutant *PPM1D* alleles. The parental HCT116 cells (*PPM1D*$^{+/mut}$) were mixed with a second, independent isogenic HCT116 (*PPM1D*$^{+/+}$) line at 1:10 ratio, and the mixed cells were cultured under the standard culture condition (split whenever a confluence was reached). Fraction of mixed cells was taken at each indicated time point for genomic DNA preparation and mutant allele quantification.

Fig. S1

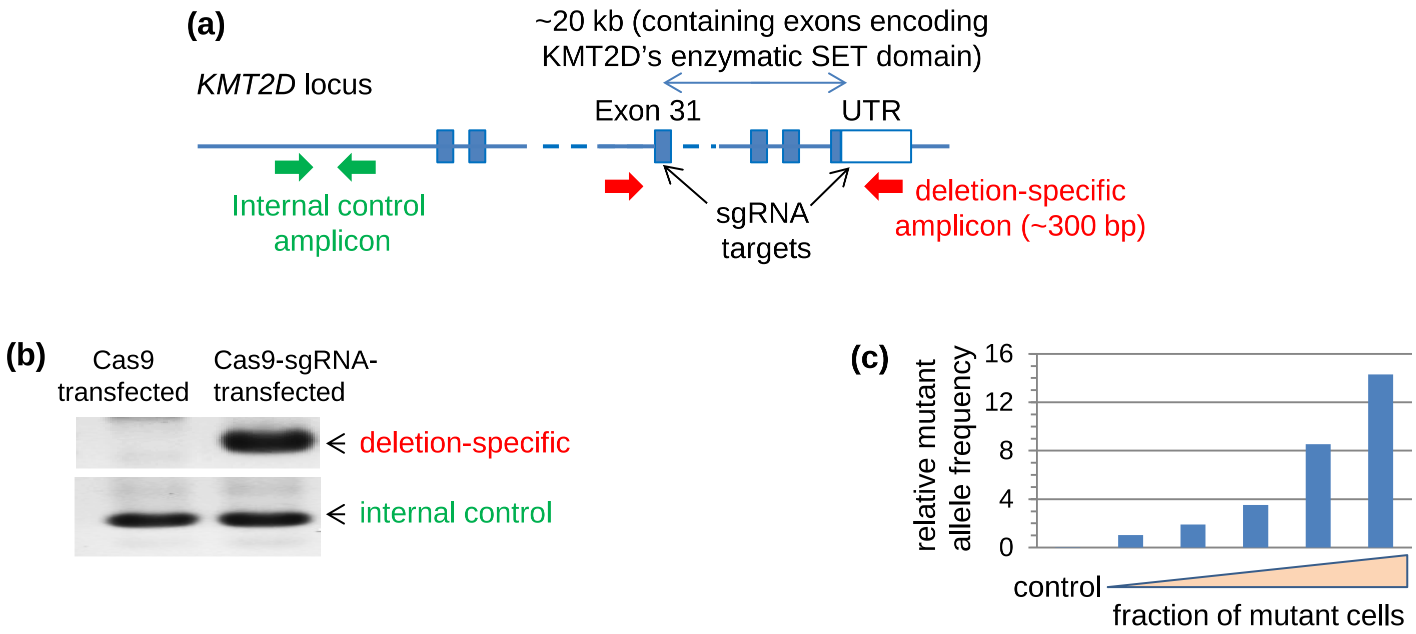

Fig. S2. Design of CRISPR-mediated *KMT2D* knockout. (a) The locations of two sgRNA (sgRNA-E31 and sgRNA-UTR) and primers used for GE-MAQ. **(b)** Detection of deletion-specific amplicon in HEK293 cells. Primers used are color-coded and shown in (a).
(c) Cas9-sgRNA-transfected HEK293 cells (containing a subset of deletion allele-carrying cells) were mixed with non-transfected cells with increasing ratios (6.25%, 12.5%, 25%, 50% and 100% of transfected cells). gDNA was prepared for determining relative deletion allele frequencies.

Fig. S2

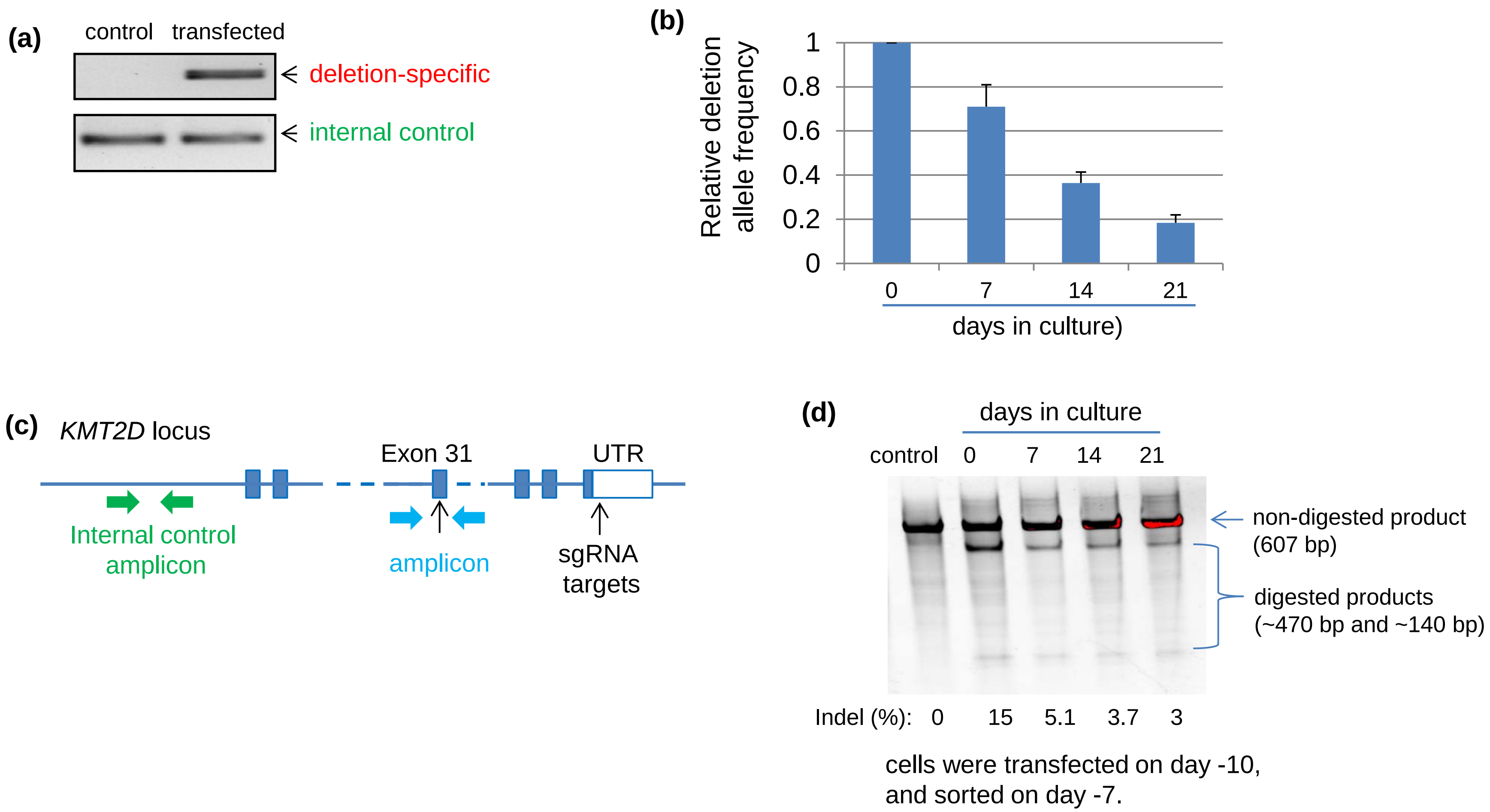

Fig. S3. *KMT2D* inactivation abrogates propagation of prostate cancer cells. (a) Validation of CRISPR-mediated *KMT2D* deletion in LAPC-4 cell line. Primers used are shown in fig. S2(a) and supplemental table 1. **(b)** GE-MAQ analysis for LAPC-4 cell lines. Note the depletion of alleles with designated deletions from the population. Experimental schematics is shown in Fig. 2B with minor modifications (cells were transfected on day 10 and sorted on day 7). **(c)** An amplicon flanking sgRNA-E31 (in blue) is designed to genotype alleles bearing no designated deletion but being affected by sgRNA-E31 (e.g, small indels). **(d)** Surveyor analysis to detect the relative abundance of mutant alleles bearing no designated deletions in LNCaP cell line. Experimental schematics were the same as (b) and primers used were colored in blue as shown in (c). Cells were transfected on day 10 and sorted on day 7.

Fig. S3

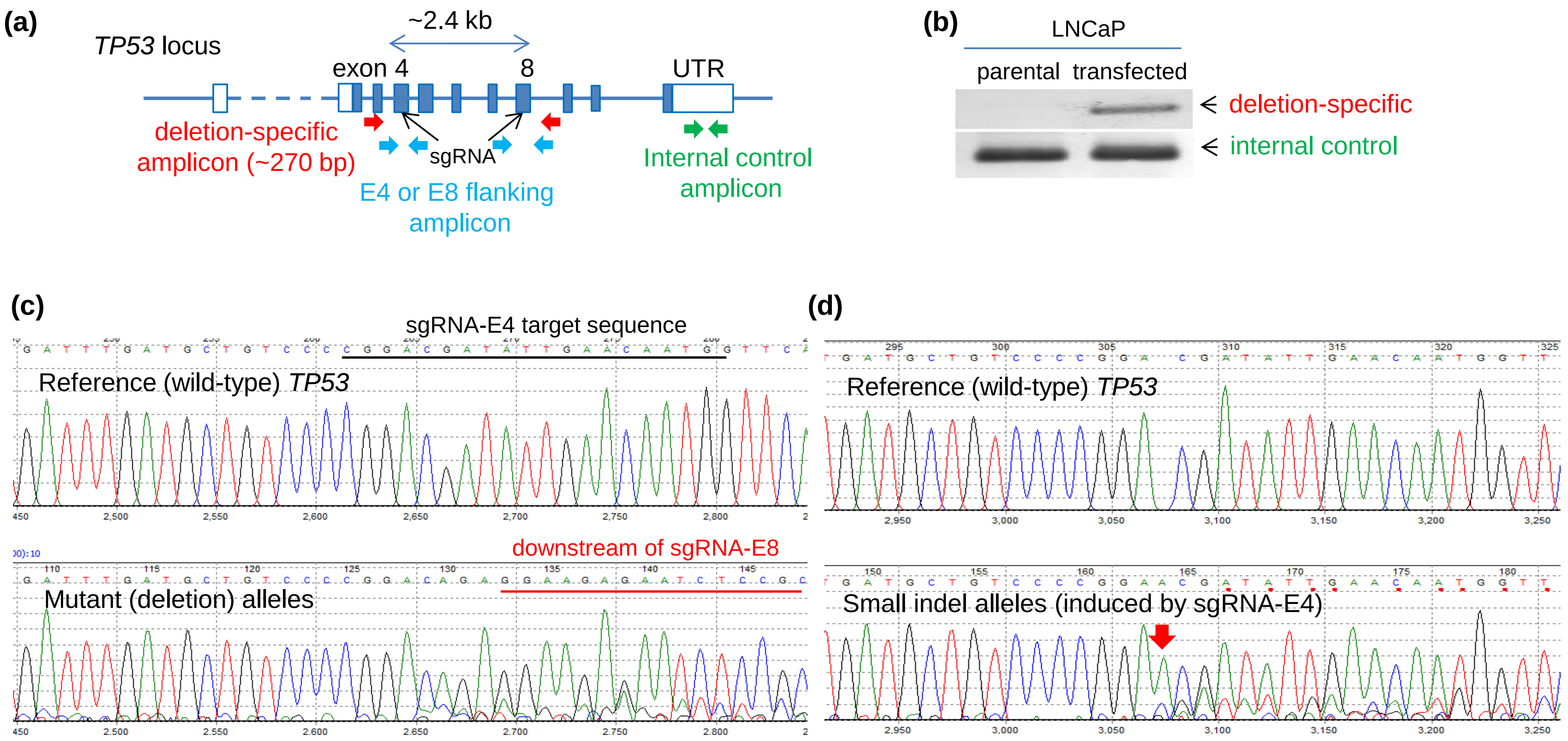

Fig. S4. Design of CRISPR-mediated *TP53* knockout. (a) Design of sgRNA-E4 and sgRNA-E8 for targeting TP53. Primers for amplifying the deletion-specific amplicon, individual sgRNA-flanking amplicons, and internal control amplicon for deletion allele's relative abundance are shown and color-coded. **(b)** PCR detection of deletion-specific amplicon in LNCaP cells one week after transfected with the pair of sgRNA CRISPR constructs. Note the heterogeneous nature of the deletion although a predominant genotype is visible. **(c)** Sequencing of the deletion-specific amplicon from the transfected LNCaP population confirms the presence of heterogeneous deletion alleles. **(d)** Mutant alleles (e.g., small indels, without designated deletion) generated by sgRNA-E4 from the heterogeneous population about 10 days after CRISPR transfection. Note one allele with an A insertion is visible (dupA allele became dominant among those without designated deletion eventually, as shown in fig. S6a).

Fig. S4

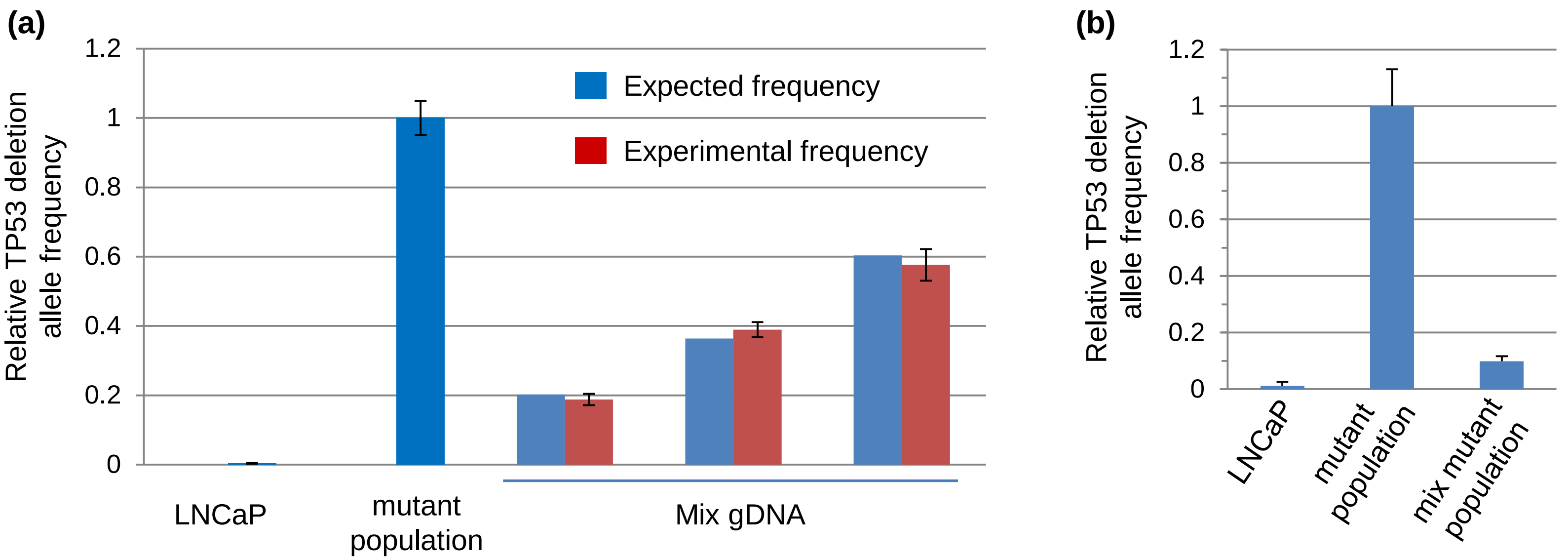

Fig. S5. Validation of qPCR method used for relative quantification of deletion allele of *TP53*. (a) Genomic DNA from the parental LNCaP and the mutant population was quantified by qPCR, and the two gDNAs were mixed at three different ratios and the expected relative frequencies were determined. The experimental allele frequencies were determined by data obtained from qPCR amplification of the deletion allele-specific amplicon. **(b)** The parental LNCaP cells, the mutant population, and their mix cell population (mutant: parental =1:9) were used for genomic DNA extraction. Relative deletion allele frequency was determined by qPCR and normalized by the internal control amplicon.

Fig. S5

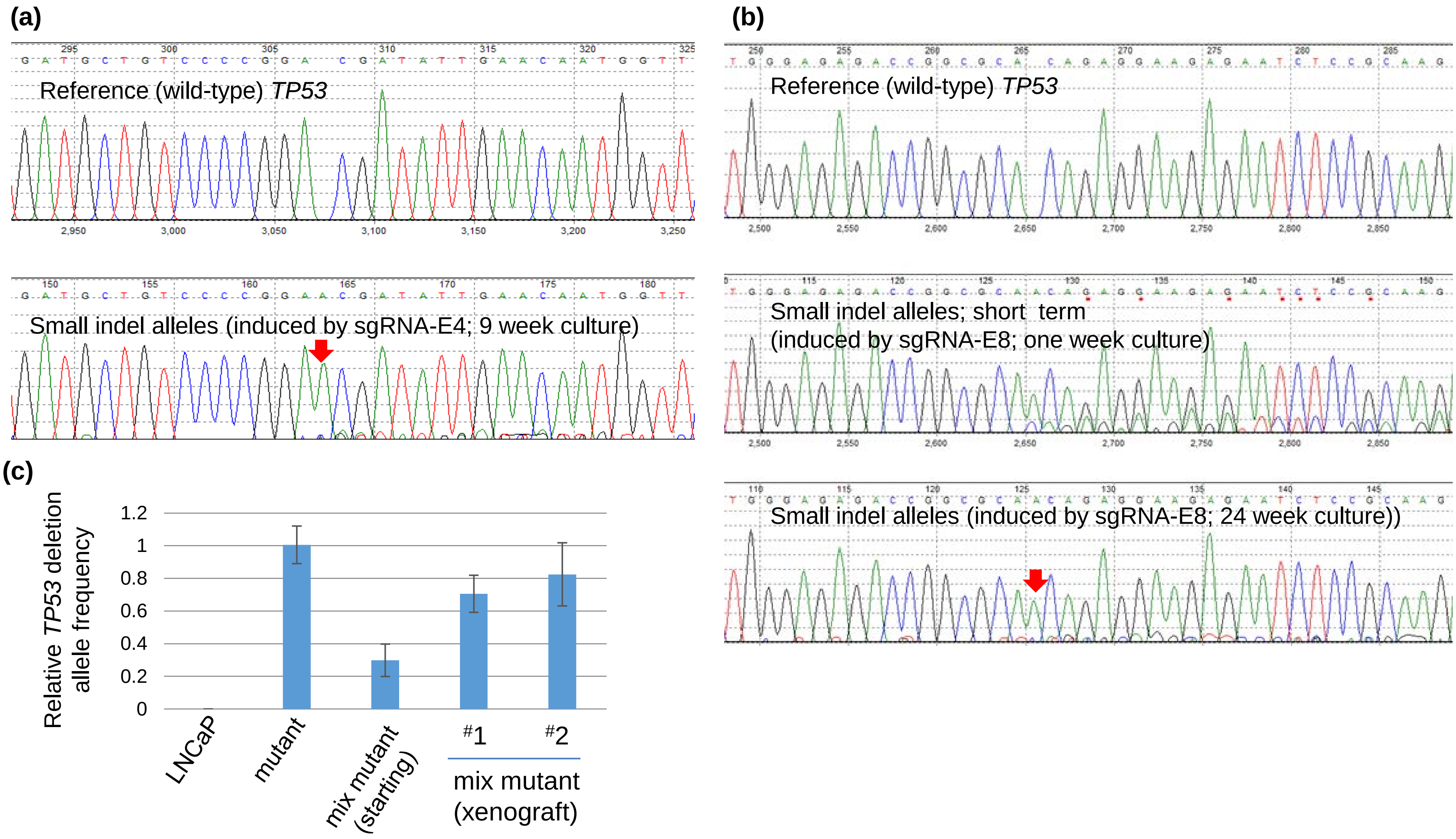

Fig. S6. *TP53* inactivation (e.g., via small indels) provides an advantage to host cells under the standard FBS-supplied culture condition and in vivo. (a) Sequencing of amplicon spanning sgRNA-E4 reveals a dominant inactivating dupA alleles (leading to amino acid change of D48fsX51) after 9 weeks of culture (note this dupA allele was emerging at the earlier stage, as shown in fig. S4d). **(b)** Similar positive selection of inactivating small indel mutations (in this case the dominant allele leads to amino acid change T284fsX305) was also observed in regions surrounding sgRNA-E8. **(c)** Mixed mutant LNCaP cells (starting; containing ~20% mutant cells) were implanted and allowed to grow in vivo for three weeks. Tumors (xenograft #1 and #2) were harvested for gDNA extraction and quantification of *TP53* deletion allele frequency. Parental LNCaP and *TP53*-null (mutant) samples were used as controls in the qPCR.

Fig. S6

(a) **(b)** **(c)**

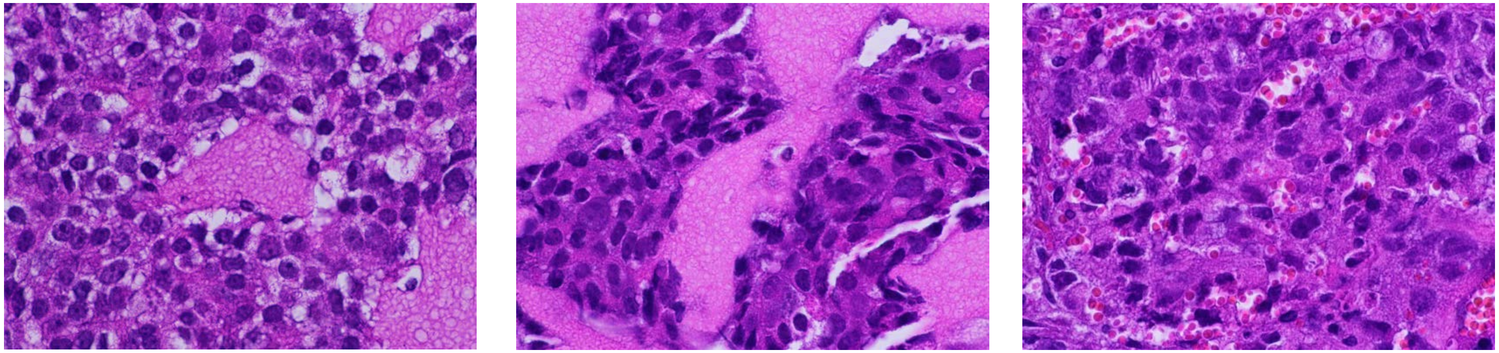

(a) Mix mutant: LNCaP parental and *TP53* mutant mix

(b) LNCaP (parental)

(c) LNCaP (*TP53* mutant)

Fig. S7. Histological images of xenograft tumors. H&E staining images (20X) of tumors derived from (**a**) mixture of LNCaP parental and *TP53* mutant cells (~20% of tumor cells were TP53 mutant)**,** **(b)** LNCaP parental cells, and **(c)** *TP53* mutant cells. At the time of tumor harvest, the sizes of these tumors were roughly 42, 37, and 64 mm^3, respectively.

Fig. S7

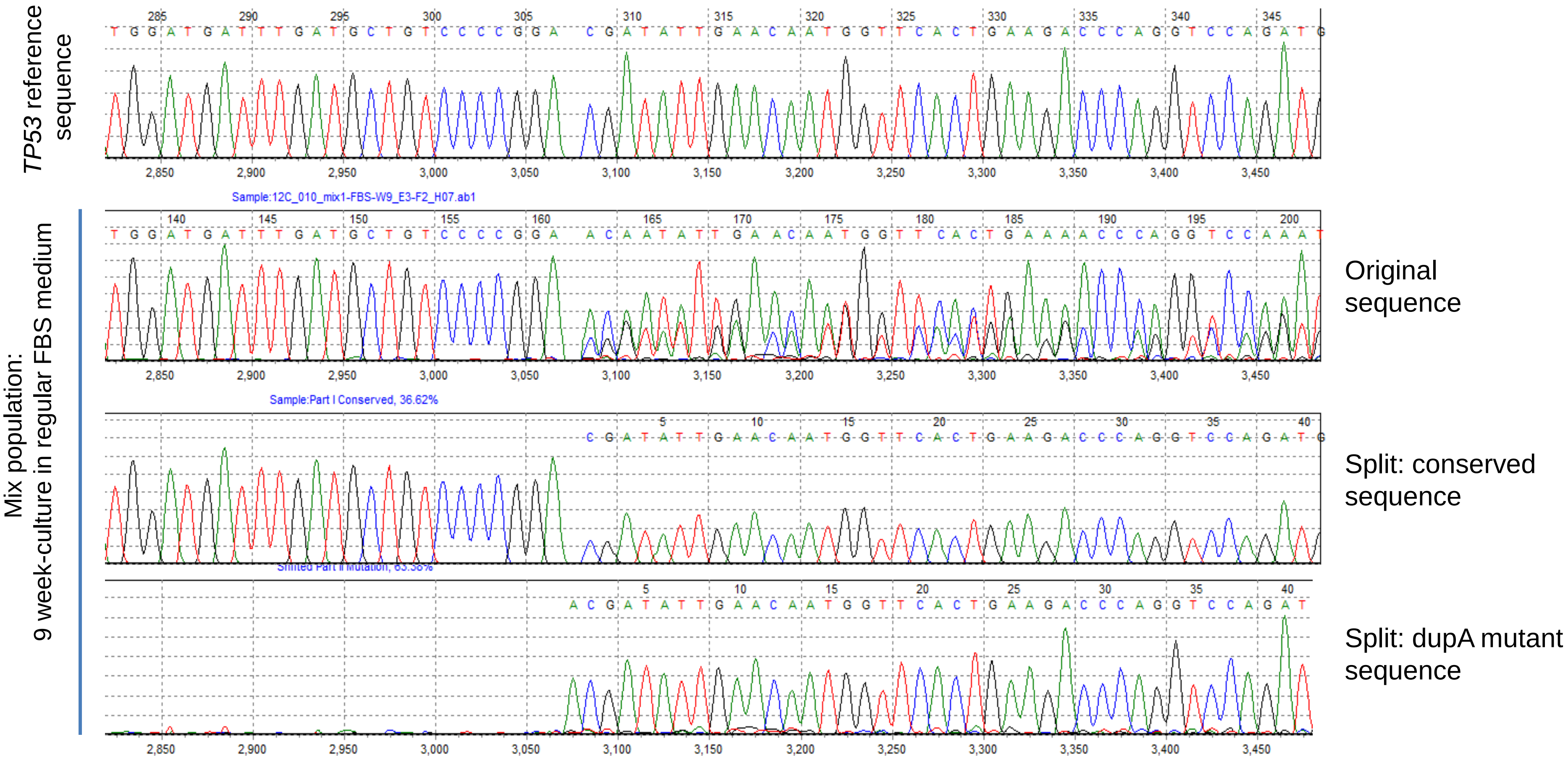

Fig. S8. Sanger sequencing analysis of heterogeneous TP53 allele. Split sequences showing the genotypes from the mix population from the 9-week culture in regular FBS media (the original sequence was also shown in Fig. 3D)

Fig. S8

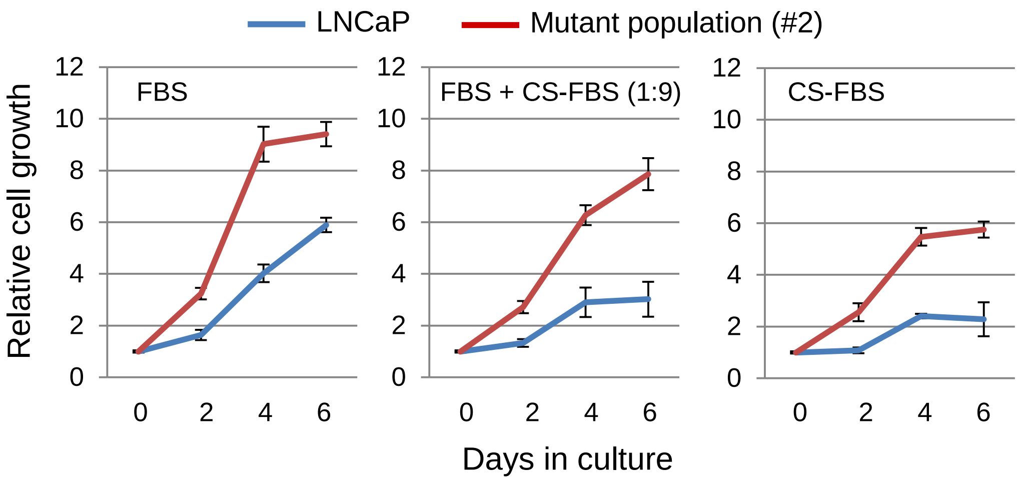

Fig. S9. TP53 inactivation provides an advantage to host cells under castration conditions. Proliferation of the parental LNCaP cells and the TP53 mutant population #2, which was generated by an independent transfection of sgRNA#1/sgRNA#2, in different medium conditions as measured by a standard cell growth assay (via cell counting kit 8) in a 96-well plate.

Fig. S9

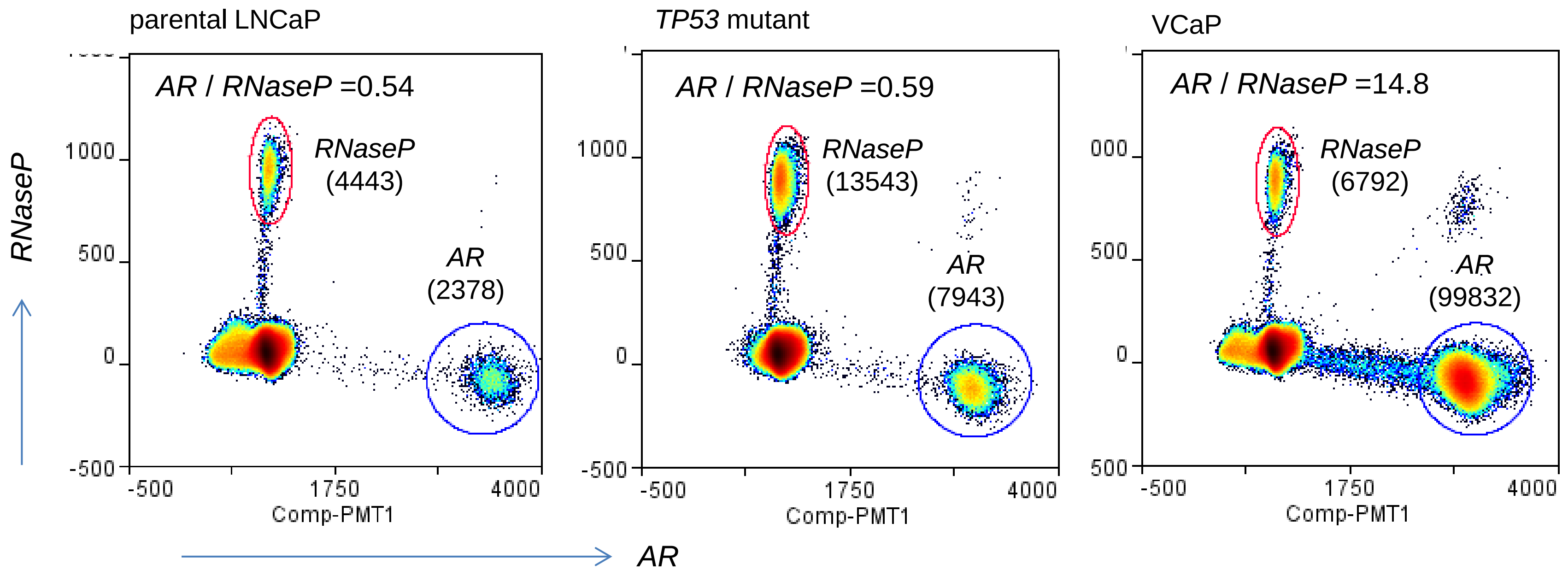

Fig. S10. Digital PCR analysis of relative AR copy numbers. gDNA from the parental LNCaP and the TP53 mutant population was used for digital quantification of AR copy numbers. RNaseP gene was used as the internal control. gDNA from VCaP, which has AR amplification, was used as a positive control.

Fig. S10

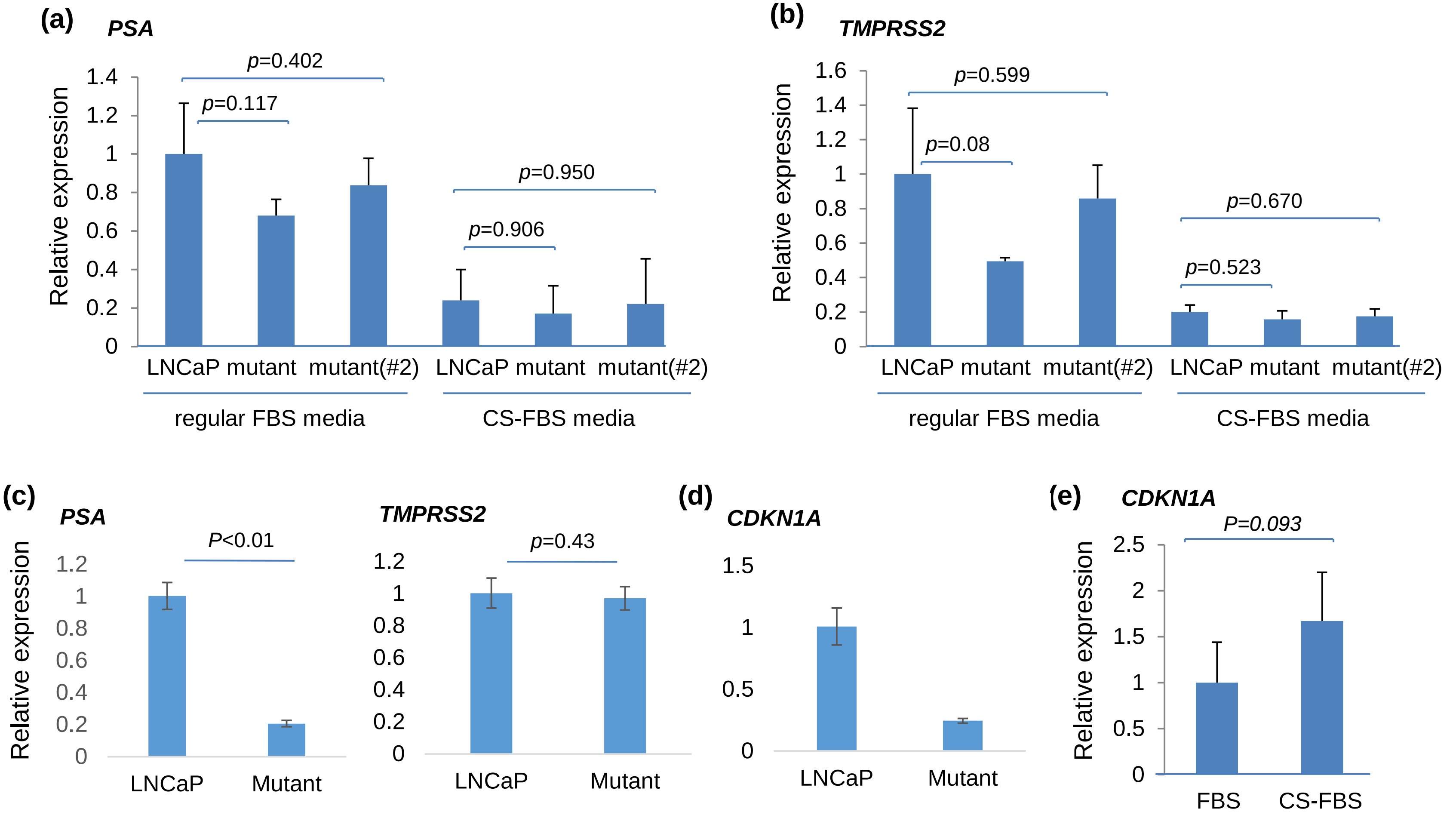

Fig. S11. Loss of p53 does not potentiate AR-mediated transcription of *PSA* and *TMPRSS2*. (a-b) Cells were placed out in regular FBS medium. After two days, the medium was replaced with fresh, regular FBS medium or with CS-FBS-supplied media; and 24 hours later RNA was isolated for RT-qPCR analysis of expression of (**a**) *PSA* and (**b**) *TMPRSS2* (HPRT was used as the internal control gene). Relative expression level of (**c**) *PSA*, *TMPRSS2* and (**d**) *CDKN2A* (measured by RT-qPCR) from LNCaP and *TP53* mutant tumors from xenografts in vivo. **(e)** The parental LNCaP cell line was placed out in regular FBS medium. After two days, the medium was replaced with fresh, regular FBS medium or with CF-FBS-supplied castration medium; and 24 hours later RNA was isolated for RT-qPCR analysis of *CDKN1A* gene expression (HPRT was used as the internal control gene).

Fig. S11

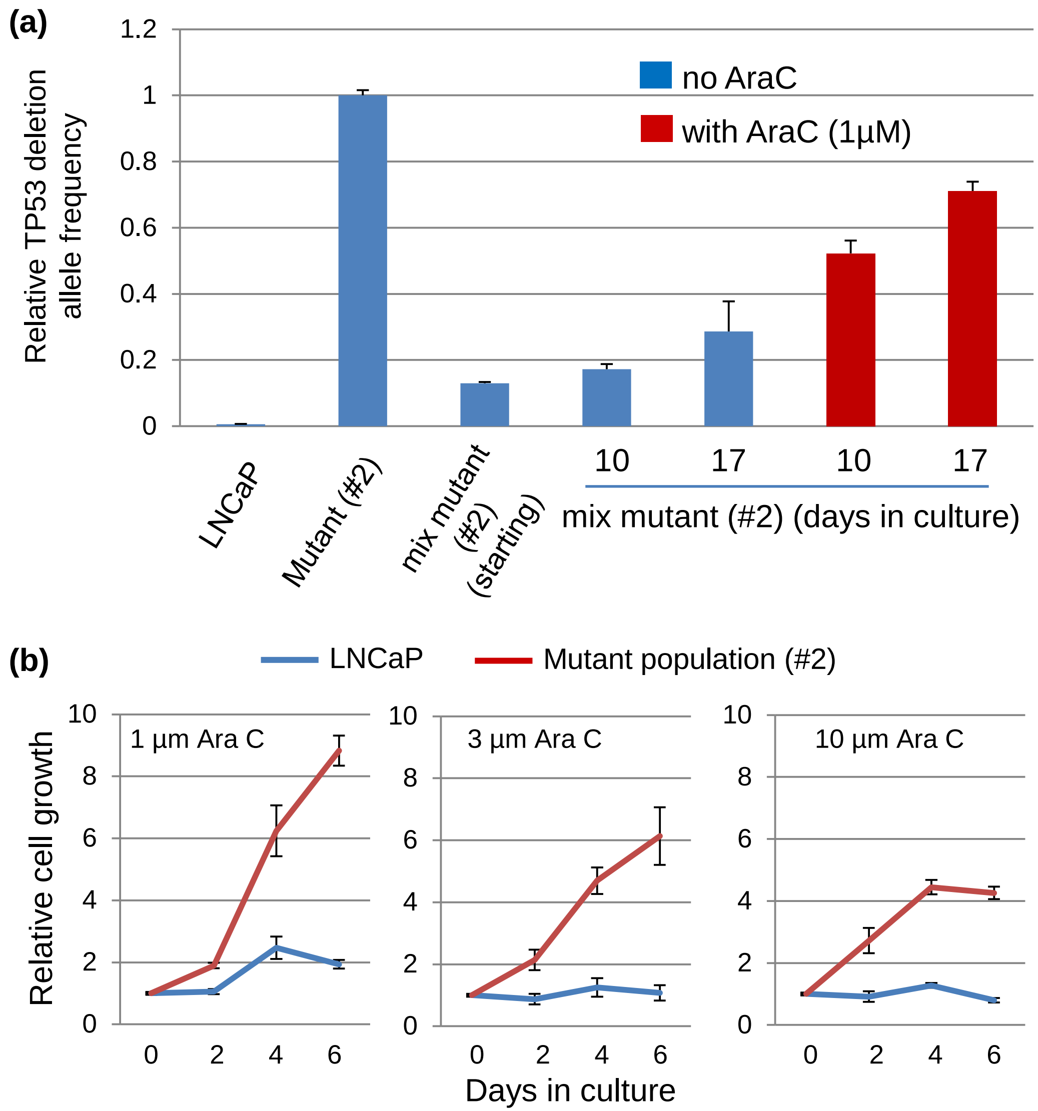

Fig. S12. ***TP53*** **inactivation provides an advantage to host cells in the presence of Ara C.** **(a)** An independently generated TP53 mutant population (mutant #2) was mixed with the parental LNCaP population (termed mix mutant, in which mutant made up 10% of total population). The mix mutant population was maintained in regular FBS-supplied media (no castration) without or with the presence of Ara C (split whenever a confluence was reached). Fraction of mixed cells was taken at each indicated time point for genomic DNA (gDNA) preparation and mutant allele quantification. **(b)** Proliferation of the parental LNCaP cells and the TP53 mutant population #2 in regular FBS-supplied media in the presence of various Ara, as measured by a standard cell growth assay (via cell counting kit 8) in a 96-well plate.

Fig. S12

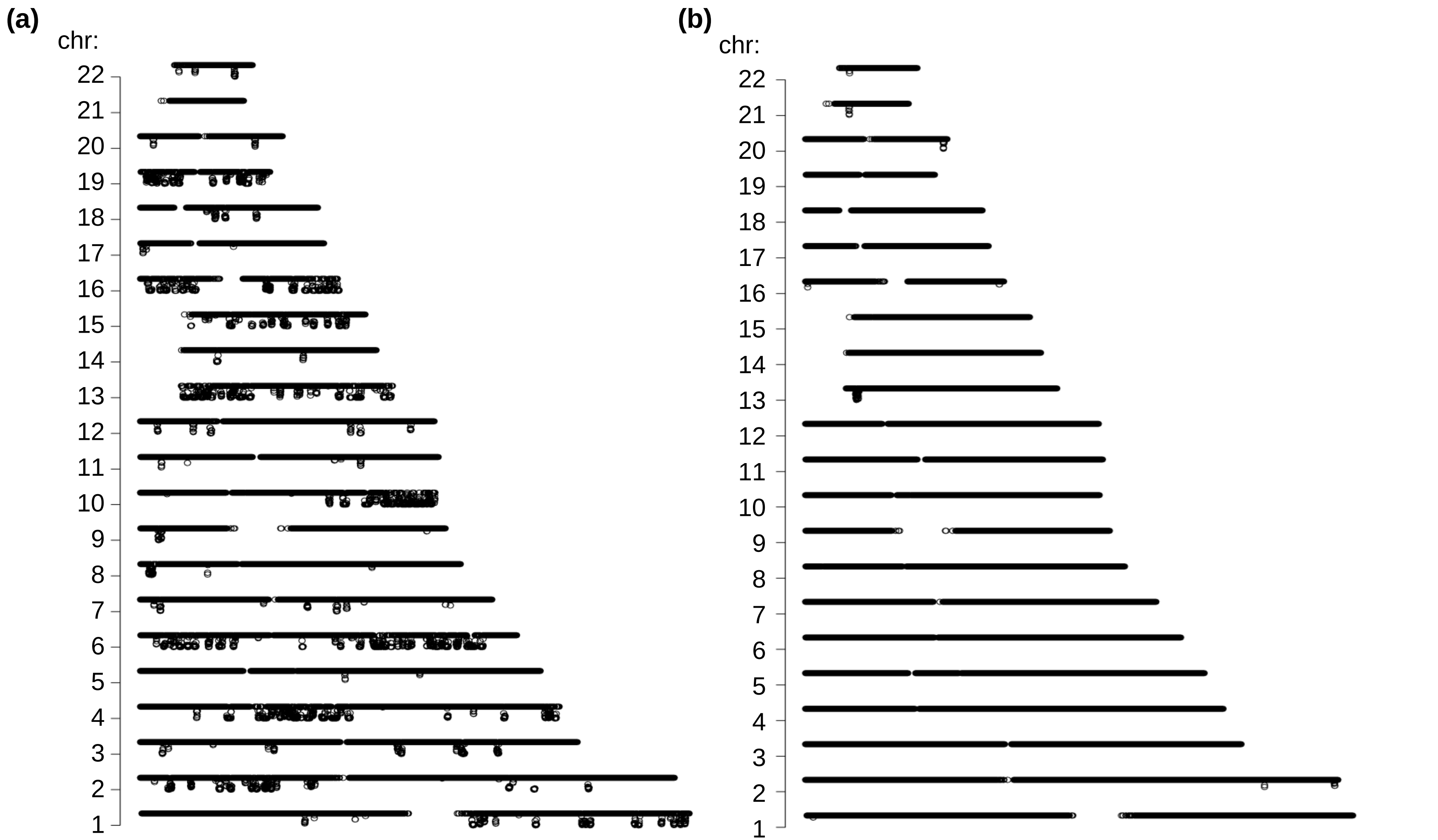

Fig. S13. ***TP53* knockout facilitates occurrence of CNVs.** CNV profiles across the genome in (**a**) the *TP53* mix mutant population that has been cultured in the standard medium in the presence of 1 µM Ara C for 14 weeks and (**b**) the initial *TP53* mutant population (24 week culture). Each sample was compared to the parental LNCaP cell line which serves as the baseline control.

Fig. S13

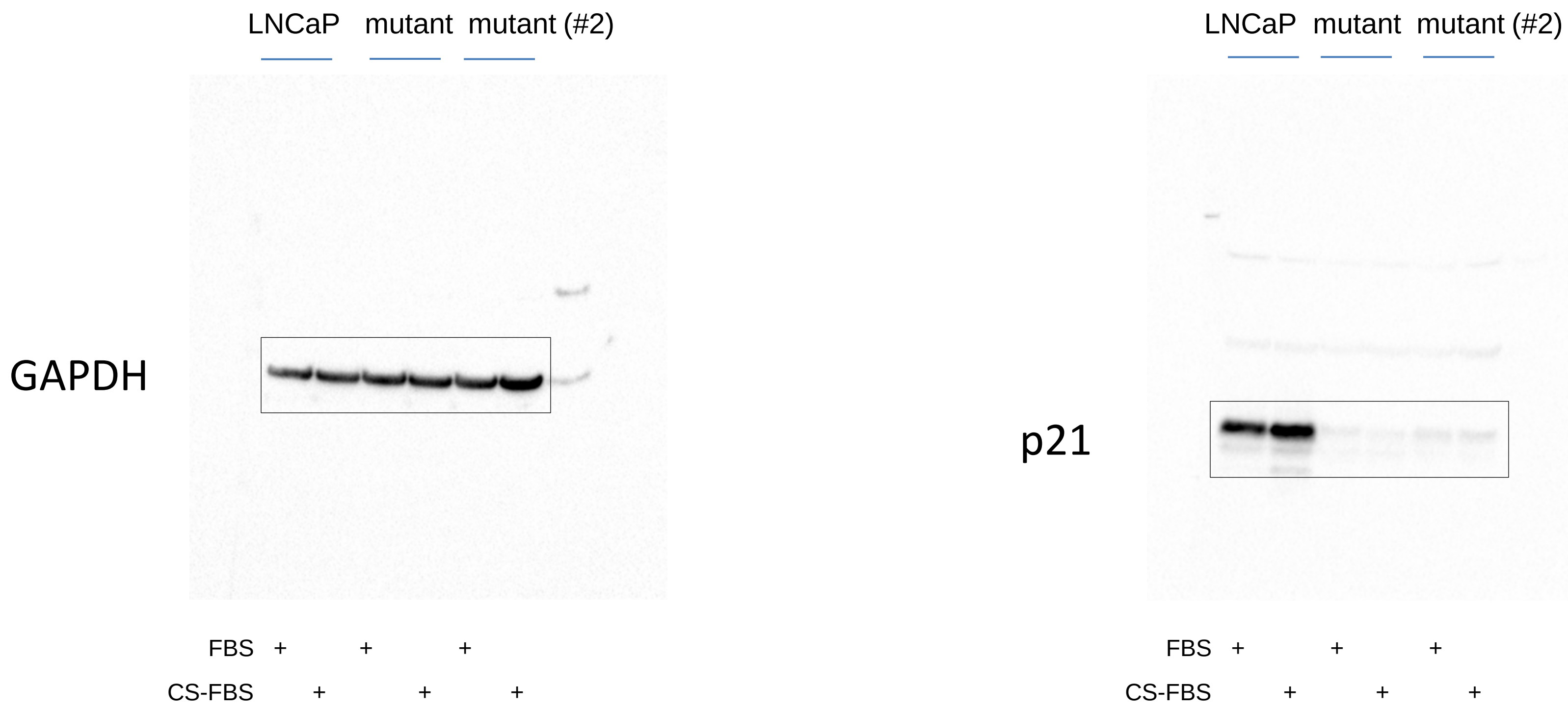

Fig. S14. Full-length western blot gels shown in Figure 4(C).

Fig. S14
